# A TCR-like CAR Promotes Sensitive Antigen Recognition and Controlled T-cell Expansion Upon mRNA Vaccination

**DOI:** 10.1158/2767-9764.CRC-21-0154

**Published:** 2022-08-18

**Authors:** Matthias Birtel, Ralf-Holger Voss, Katharina Reinhard, Benjamin Rengstl, Yasmina Ouchan, Kristina Michel, Nina Hayduk, Bodo Tillmann, René Becker, Martin Suchan, Matthias Theobald, Petra Oehm, Özlem Türeci, Ugur Sahin

**Affiliations:** 1 TRON – Translational Oncology at the University Medical Center of the Johannes Gutenberg University gGmbH (non-profit), Mainz, Germany.; 2 Biopharmaceutical New Technologies (BioNTech) Corporation, BioNTech Cell & Gene Therapies GmbH, Mainz, Germany.; 3 Department of Research Center for Immunotherapy (FZI), University Medical Center (UMC) of the Johannes Gutenberg University, Mainz, Germany.; 4 Department of Hematology, Oncology, and Pneumology, University Cancer Center (UCT), University Medical Center (UMC) of Johannes Gutenberg University, Mainz, Germany.

## Abstract

Chimeric antigen receptor (CAR) T cells are efficacious in patients with B-cell malignancies, while their activity is limited in patients with solid tumors. We developed a novel heterodimeric TCR-like CAR (TCAR) designed to achieve optimal chain pairing and integration into the T-cell CD3 signaling complex. The TCAR mediated high antigen sensitivity and potent antigen-specific T-cell effector functions in short-term *in vitro* assays. Both persistence and functionality of TCAR T cells were augmented by provision of costimulatory signals, which improved proliferation *in vitro* and *in vivo*. Combination with a nanoparticulate RNA vaccine, developed for *in vivo* expansion of CAR T cells, promoted tightly controlled expansion, survival, and antitumor efficacy of TCAR T cells *in vivo*.

**Significance:**

A novel TCAR is tightly controlled by RNA vaccine–mediated costimulation and may provide an alternative to second-generation CARs for the treatment of solid tumors.

## Introduction

Adoptive cell therapy (ACT) with autologous T lymphocytes genetically engineered to express chimeric antigen receptors (CAR) is an effective treatment for patients with B-cell malignancies. However, this technology has proven less effective in treatment of solid tumors. Three of the main challenges for CAR T-cell therapies in solid tumors are antigen escape, limited CAR T-cell persistence and CAR-mediated on-target or off-target toxicities. Hence, there is an unmet need for a CAR T-cell therapy that is effective in this context.

Similar to T-cell receptors (TCR) which bind to antigen peptide–loaded MHC molecules, CARs mediate strong interaction of modified T cells with surface antigens on hematologic or solid tumors, allowing efficient killing of malignant cells. The CAR T-cell products available on the market for clinical use bear second-generation CAR scaffolds that differ substantially from the initial CAR designs described in the 1990s. The original CARs were composed of antigen-binding moieties hooked onto the TCR constant alpha- and beta-chain (Cα and Cβ). These emulated the endogenous TCR by recruiting CD3, the activation machinery required for proper surface expression and function (1, 2). The so-called first-generation CAR design on the other hand consisted of the antigen-binding single-chain variable fragments (scFv) directly fused to CD3 subunit CD3ζ, bypassing the need to recruit the entire endogenous CD3 complex (3). In patients, however, activation, survival, and persistence of CAR T cells equipped with first-generation CARs were poor. These shortcomings were addressed by incorporating costimulatory domains derived from the T-cell activation machinery into the CAR designs: second-generation CAR constructs feature a single costimulatory domain (4, 5), while third-generation CARs contain two costimulatory domains (6). The combination of costimulatory domains CD28 or 4-1BB with CD3ζ signaling in second-generation CAR formats (CAR-28ζ; CAR-BBζ) has been used successfully in the clinic (7). However, third-generation CAR formats have so far not led to any further improvements.

The endogenous TCR/CD3 complex is a multiprotein composite of eight subunits whose activation is tightly and reversibly regulated (8–10). Along the evolution of CAR designs, the concept of fusing CD3ζ into the single chain had been introduced while the physiologic signaling through the TCR/CD3 complex was abandoned. Instead, second-generation CAR formats hijack the finely-tuned T-cell signaling machinery, which results in the formation of nonclassical immunologic synapses (11). In comparison with endogenous TCRs, CAR-28ζ have been shown to have lower sensitivity in recognition of their target and reduced signaling capacity (12), which leads to the hypothesis that signaling through the CD3 complex could avoid antigen low or negative tumor relapse in the clinics (13). In this context, previous studies on TCR function found that the full set and quality of the 10 immunoreceptor tyrosine-based activation motifs (ITAM) in the TCR/CD3 complex is important for TCR signaling and robust and scalable T-cell function (14). This might also explain some of the observed shortcomings of some second-generation CAR scaffolds, which rely exclusively on the three ITAM motifs of CD3ζ. In addition to the ITAM quality and multiplicity, the formation of presynaptic TCR clusters has also been associated with TCR sensitivity and function (15, 16).

We hypothesized that in particular for a CAR treatment of solid tumors, the lack of cognate antigen encounter in the periphery and low antigen expression in the tumor lesion might favor receptors with physiologic signaling and higher antigen sensitivity. To test this hypothesis, and using our previous experience in TCR design (17), we developed a TCR-like CAR (TCAR) designed to more closely mimic the dimeric TCR structure and physiologic signaling pathway. Similar to previous designs, the scFv domains were fused to Cα and Cβ. In contrast to other CAR designs, however, we arranged identical variable domains in tandem on either chain (VH-VH-Cα/VL-VL-Cβ) to enhance dimerization of the construct via the variable domains (18, 19) and optimize integration into a synthetic TCAR/CD3 complex. We postulated that enhanced dimerization may allow us to use weakly interacting human instead of strongly interacting murine TCR C-domains.

For proof-of-concept studies, we engineered the TCAR to target two well-characterized and safe tumor-associated antigens from the Claudin family of tight junction proteins, Claudin 6 (CLDN6) and Claudin 18.2 (CLDN18.2). Both have previously been deemed safe for CAR-based therapies due to their cancer tissue specific expression (20, 21). Antibody and CAR therapies against both targets are currently in clinical development, and the antigen-specific variable domains selected for TCAR development are based on those existing designs. The CLDN6-specific variable domains are highly related to those of IMAB027, a clinical-stage IgG1 mAb [(22); NCT02054351, (23)] and used in a second-generation CAR that is currently being evaluated in patients with CLDN6-positive relapsed or refractory advanced solid tumors (NCT04503278). The CLDN18.2-specific variable domains originate from the CLDN18.2-specific antibody Zolbetuximab (previously IMAB362). This antibody is in advanced clinical development for the treatment of gastric and pancreatic cancers (24), while CLDN18.2 is also being targeted by second-generation CAR-based therapy in clinical trials (NCT04404595, NCT04966143, NCT03890198, NCT03874897).

As the TCAR was designed to recruit CD3, and signal in a TCR-like fashion, it was similarly expected to undergo only limited antigen-dependent proliferation against tumor cells, which are naturally lacking in costimulatory molecules. We therefore provided costimulatory signals by applying our recently described CAR-T cell Amplifying RNA-lipoplex vaccine (referred to as CARVac) technology *in vivo*. CARVac is based on the systemic administration of RNA lipoplexes (RNA-LPX) encoding the full-length CAR target antigen, which has previously been shown to provide costimulus to CAR-engineered T cells (20, 25).

We first optimized the TCAR design, tested chain pairing and recruitment of T-cell endogenous CD3 by different formats *in vitro* with RNA-transfected cells. The lead candidate was then developed further and retrovirally transduced into T cells, to characterize the receptor in long-term *in vitro* experiments and also test proliferation, expansion, and antitumor activity in mice *in vivo* in combination with the CARVac technology. This work furthers our understanding of CAR design and function and provides a new tool for the development of effective therapies to treat solid tumors.

## Materials and Methods

### TCAR Design

The goal of this study was to identify a CAR design, which uses the T-cell endogenous CD3 complexes for T-cell signaling and function. Two well-established tumor-associated target antigens of the Claudin family (CLDN6 and CLDN18.2) were used as CAR target antigens. Variable domains of IMAB206-C46S (WO20121560018) or IMAB362 (WO2013174404A1) were applied for the generation of CLDN6- or CLDN18.2-specific CARs, respectively. The CLDN6-specific variable domains are highly related to IMAB027, a therapeutic antibody against CLDN6 (22), while Zolbetuximab [previously IMAB362 (24)] was taken for CLDN18.2-specific receptor generation. FMC63 was used for the generation of CD19-specific CARs. scFv fragments were cloned by fusion of variable domains in indicated orientation connected via glycine-serine rich linkers. Several different TCR-like CARs (referred to as TCAR) were generated by fusing antibody variable domains, and chimerized to the constant domains of the mouse or human TCRα or TCRβ chain via a short gagq containing sequence. An artificial disulfide bond between Cα and Cβ was introduced at position hTRAC T47C (Uniprot: P01848) or mTRAC T48C (UniProt: A0A075B662) and hTRBC S57C (UniProt: P01850) or mTRBC [UniProt: A0A075B5J4 (26)]. For the incorporation into retroviral backbones, both the CLDN6- and CLDN18.2-TCAR chains were linked via self-processing 2A-elements (27) on a single construct [VL-VL-Cβ-P2A-VH-VH-Cα (28)] and were subcloned into the self-inactivating retroviral vector pES12.6 with the orientation VL-VL-Cβ-P2A-VH-VH-Cα (29). CAR-BBζ and CAR-28ζ constructs were used as second-generation reference constructs (20, 30). The CAR-BBζ construct was designed as described previously (20). For a CAR-28ζ, a previously published scaffold was used [Uniprot: P10747, hCD28 with mutations P208A, P211A, P212A (30)]. To perturb antigen binding of CLDN6-specific CAR constructs, site-specific mutations of CDR3 regions in VH domains were performed (XL QuikChange site-directed mutagenesis, Agilent). The CDR3 region of the heavy chain variable domain was identified using the IMGT database (31). According to IMGT database, nomenclature mutations were introduced in VH at position F113A. A murine CLDN6-specific TCR was used (WO20121560018) as a TCR control. For Myc-tagged immunoreceptors, two Myc-tags were fused in tandem N-terminally to the receptors’ variable domains.

Codon-optimized receptor and antigen sequences were synthesized by MWG Eurofins and subcloned into vectors for RNA production or retroviral vectors. For *in vivo* bioluminescence imaging experiments, the receptors were combined with 2A-linked firefly luciferase and enhanced GFP (eGFP) reporter genes.

### RNA Production and Retroviral Supernatants

The vectors pST1-T7-GG-hAg-MCS-2hBg-A30L70 or A120 were used as in vitro transcribed (IVT) RNA production templates (human α-globulin, two 3′ human β-globulin untranslated region (UTR) and a poly(A) tail containing 30 + 70 adenosine residues separated by a 10 nucleotide linker or a continuous 120 adenosine sequence). The 5′ and 3′ UTRs and poly(A) tails have been previously optimized for stability and protein translation (20, 32). IVT RNA production and simultaneous capping with β-S-anti-reverse cap analog were performed as previously described from linearized DNA templates (33). GALV and MLV-E pseudotyped retroviral supernatants for transduction of human and murine T cells were produced by transfection of 293Vec-Galv and Platinum-E cell lines with the receptor encoding self-inactivating retroviral vector pES12.6 (29).

### Animals

NOD.Cg-*Prkdc^scid^ Il2rg^tm1Wjl^/SzJ* (NSG) breeding pairs were purchased from Jackson laboratory. Age and sex mixed siblings were used for the xenograft experiment. Female BALB/c or C57BL/6BrdCrHsd-*Tyr^c^* mice (Envigo and Janvier) were purchased, while C57BL/6-Thy1.1 and BALB/c-Thy1.1 donor mice (Jackson Lab) were bred in the animal facility of BioNTech SE, Germany. All animal experiments were performed under specific pathogen-free conditions and according to German animal ethics experimentation regulations.

### Cell Lines and Human Primary Cell Culture

Cell culturing was performed following standardized protocols. After thawing, cell lines were cultured for a maximum of 6 weeks. The following cell lines were acquired commercially in the corresponding years: CT26 (ATCC, 2016), NCI-87 (ATCC, 2018), PA-1 (ATCC, 2016), SK-OV-3 (ATCC, 2014), Colo-699-N (Sigma-Aldrich, 2018), and Platinum-E (BioCat, 2018). Cell line authentication was performed on working cell banks while *Mycoplasma* testing was performed both on main and working cell banks using PCR technology. Testing was outsourced to Eurofins Genomics. PA-1-SC12_A02_luc_egfp_CLDN6^+^ and PA-1_A02_gfp_CLDN6^−/−^_CLDN18.2^+^ (referred to as PA-1^CLDN6+^ or PA-1^CLDN18.2+^, respectively) are derivatives of a single-cell clone of the human endogenously CLDN6-expressing teratoma cell line PA-1 lentivirally transduced to overexpress HLA-A*0201 and GFP and were generated as described previously (20). CT26 and NCI-N87 were transduced with mouse or human CLDN18.2 encoding lentiviruses, respectively [CT26^CLDN18.2+^ and NCI-N87^CLDN18.2+^ (20)]. For generation of GALV and MLV-E pseudotyped viral particles 293Vec-Galv (34) and Platinum-E cell lines were used. Cells were transfected with respective receptor encoding retroviral transfer vector pES12.6 using TransIT-LT1 (Mirus) according to the manufacturer's instructions. Retroviral supernatants were collected 48 and 72 hours after transfection. Titer determination was performed with Jurkat cells, parental or stably transfected with mCAT-1 (35) retroviral transductions were adjusted to 10^6^ infectious units/mL for each supernatant.

Peripheral blood mononuclear cells (PBMC) were isolated by Ficoll-Paque PLUS (Amersham Biosciences) density gradient centrifugation from buffy coats from healthy donors. For immature dendritic cell generation (referred to as DC), CD14^+^ cells were enriched using magnetic-activated cell sorting (MACS) technology (Miltenyi Biotec). CD14^+^ cells were differentiated toward DCs by culture media consisting of RPMI1640 GlutaMax supplemented with 5% (v/v) pooled human serum (PHS, OneLambda), 1 mmol/L sodium pyruvate, nonessential amino acids and 1,000 IU/mL rhGMCSF and rhIL4 (both Miltenyi Biotec) over 5–7 days, reported previously (20). CD14^+^ cell–depleted PBMCs were subjected to MACS-mediated cell separation or frozen for proliferation assays with receptor-transfected T cells. Frozen PBMCs were thawed one day prior to coculture establishment, the respective T-cell population was isolated using MACS technology and electroporated with RNA. Each experiment using DCs was performed in the autologous setting.

### Flow Cytometry

The following antibodies and fluorescent dyes were used for flow cytometric analysis. hCD3ε-FITC (UCHT1, BD), hCD4-APC-Cy7 (OKT4, BioLegend), hCD4-PE (RPA-T4, BD), hCD8α-FITC (B9.11, Beckman Coulter), mCD8α-eFluor506 (53–6.7, eBioscience), hCD8α-BV421 (RPA-T8, BD), hCD8α-APC-Cy7 (RPA-T8, BD), mCD11c-APC (N418, Miltenyi Biotec), mCD11b-APC-eFluor780 (M1/7, Invitrogen), mCD62L-APC-Fire750 (MEL-14, BioLegend), hCD80-PE, mCD90.1-PerCP (Ox-7, BD), mCD90.2-eFluor450 (53–2.1, eBioscience), mCD90.2-PE (53–2.1, BD), mCD127-PE-Cy7 (SB/199, BD), hCD137L-PE (5F4, BioLegend), mCD137L-PerCP-eFluor710 (TKS-1, eBioscience), mKLRG1-eFluor450 (2F1, Invitrogen), 7-AAD (Beckman Coulter), LIVE/DEAD fixable yellow dead cell stain kit (Invitrogen). CLDN6 and CLDN18.2-CARs were stained with an anti-idiotype specific antibody coupled to Alexa647. Anti-CLDN6-DyLight650 (WO20121560018; clone IMAB027) and anti-CLDN18.2-DyLight650 (WO2013174404A1; clone IMAB362) were used for antigen stainings. A total of 2 × 10^4^ to 5 × 10^5^ cells were stained for surface antigen expression and fixed using BD stabilizing fixative (BD). For analysis of mouse or human T cells, 40 μL peripheral heparinized blood were stained with antibodies and subsequently lysed using ACK lysis buffer (gibco). Murine DCs were identified out of spleen homogenate via CD11c and CD11b positive staining. FACS data were acquired using FACS Diva software (BD) on either Canto or Canto II (BD) systems. Data analysis was performed using FlowJo 10. Frequency of CAR T-cell numbers were calibrated on the basis of flow cytometric data.

### Electroporation of IVT RNA

CD3^+^ cells were isolated from CD14^+^-depleted PBMCs via MACS technology. Prior to electroporation, cells were washed once with ice-cold X-VIVO 15 media (Lonza). A maximum of 10^7^ cells were applied for electroporation in 4 mm electroporation cuvettes (VWR) in 250 μL X-VIVO 15 media at 4°C. Electroporation was performed either on a Cytopulse PA4000 or a BTX ECM830. If not stated differently, 4 μg Cα, 8 μg TCR chain, 12 μg TCAR chain, 15 μg CAR-BBζ, and 20 μg CAR-28ζ encoding RNAs were used. Cells were electroporated with the following conditions. Colo-699-N: 300 V, 8 ms; Jurkat76: 375 V, 10 ms; DCs: 300 V, 12 ms; hCD3^+^ T cells: 495 V, 3 ms; SK-OV-3: 200 V, 12 ms, 2 pulses. After electroporation, cells were kept in culture medium for 18–20 hours before being used in assays.

### Retroviral Transduction of Human and Mouse T Cells

Human T cells were enriched and activated from CD14^+^-depleted PBMCs on day 1 with Dynabeads Human T-Expander CD3/CD28 (Gibco; 3 beads/cell) in X-VIVO 15 media supplemented with 5% PHS in the presence of 450 IU/mL rhIL7 and 50 IU/mL rhIL15 (Miltenyi Biotec) for 3 days. On day 4, activated T cells were debeaded and incubated on GALV-pseudotyped gamma retrovirus precoated RetroNectin plates at a concentration of 10^6^ cells/mL in IL7/IL15-supplemented culture media. On day 6, cells were seeded for expansion at 0.5 × 10^6^ cells/mL in fresh media containing cytokines. Cells were either directly used on day 8 to assess receptor surface expression, T-cell phenotype and *in vitro/ in vivo* effector functions or cryopreserved.

For transduction of mouse T cells, erythrocyte-depleted splenocytes were isolated from C57BL/6-Thy1.1^+^ or BALB/c-Thy1.1^+^ mice on day 1. Cells were subsequently activated in the presence of a beads-to-CD3^+^ T-cell ratio of 1:1 using Dynabeads Mouse T-Activator CD3/CD28 (Gibco) in RPMI1640-GlutaMAX supplemented with 10% FBS, 1 × nonessential amino acids, 1 mmol/L sodium pyruvate, 10 mmol/L HEPES, 50 μmol/L β-Mercaptoethanol, 50 IU/mL Penicillin, and 50 μg/mL Streptomycin 450 IU/mL rhIL7 and 50 IU/mL rhIL15. A total of 24 hours after bead activation, cells were gently spun down (1 hour, 37°C, 300 *×* g) and incubated on MLVE-pseudotyped gamma-retroviral vector precoated-RetroNectin-plates. After additional overnight cultivation, spin-down transduction was repeated on freshly viral particles coated plates. On day 4, mouse T cells were debeaded and expanded in fresh media containing rhIL7 and rhIL15. Adoptive cell transfer was performed on day 7. To avoid the high competition of TCAR with endogenous TCRs for CD3 in mouse T cells, mouse T cells were transduced with TCAR carrying mouse C-domains constructs.

### *In Vitro* Assays

Endogenously antigen-expressing or RNA-transfected tumor cells or autologous DCs were used for functional testing of receptor-transfected CD8^+^ or transduced CD4^+^/CD8^+^ T cells. For IFNγ secretion and cytotoxicity assays, CD8^+^ T cells were activated for 3 days using plate-bound anti-hCD3ε antibody (clone OKT3; BioXcell) in the presence of 50 or 250 IU/mL rhIL2 (Novartis; Supplementary Fig. S1C) and rested for an additional 3 days. For proliferation assays, nonactivated CAR RNA-electroporated CD8^+^ or CAR-transduced CD4^+^/CD8^+^ T cells were stained with 0.8 μmol/L carboxyfluorescein diacetate succinimidyl ester (CFSE) or 1 μmol/L CellTrace Violet (Invitrogen). Dilution of the fluorescent dye over daughter generations was measured by flow cytometry as a surrogate for proliferation after 5 days of coculture. IFNγ secretion assays were performed using the kit ELISA Ready-SET-Go (Invitrogen). Absorbance was detected using a Sunrise Microplate Reader (Tecan).

Luciferase-based cytotoxicity assays were performed using preactivated electroporated CD8^+^ or transduced T cells as described previously (36). Target cells were coelectroporated with indicated amounts of antigen and 5 μg of luciferase encoding IVT RNA. The next day, transfected target cells were seeded into 96-well Nunc white plates (Thermo Fisher Scientific) and cocultured with preactivated and receptor modified T cells. After 3 hours, Triton-X100 (0.2% v/v) was added to positive control wells and HEPES (Gibco) buffered Moonlight d-luciferin (BD Biosciences) at a concentration of 12 mg/mL to all wells. Luminescence of cocultures was measured after the indicated timepoints using an Infinite M200 plate reader (Tecan). For impedance-based cytotoxicity assays, the xCELLigence (OMNI life science) system was used. Attachment of adherent tumor cells to 96-well E-plates (Agilent) was measured as an increase in impedance or cell index (CI) units over 20 hours. CI impedance measurements were performed according to the instructions of the supplier. A total of 2 × 10^4^ target cells were seeded per well in 96-well E-plates. After 20 hours, effector cells were added and cytotoxicity was determined by decrease in CI with the formula (CImax − CIsample)/CImax × 100. CAR T-cell function was assessed via reduction of CI over time. For spheroid killing assays, 10^4^ eGFP and CLDN6- or CLDN18.2-expressing PA-1 derivatives (referred to as PA-1^CLDN6+/CLDN18.2+^) tumor cells were left to form tumor spheroids for 48 hours in ultra-low attachment 96-well plates (Costar). For CD80-expressing and 4-1BBL–expressing tumor spheroids, tumor cells were electroporated and incubated for 24 hours. CAR RNA-electroporated CD8^+^ T cells were added after 24 hours, while transduced CD3^+^ T cells were added after 48 hours in FluoroBrite DMEM (Gibco) supplemented with 5% (v/v) PHS. Spheroid cultures were monitored at 4-fold magnification at an exposure time of 300 ms in an IncuCyte S3 Live-content imaging system (Essen Bioscience) at 37°C, 5% CO_2_. Total green object integrated intensity (GCU × μm^2^/Image) was assessed using the IncuCyte analysis software. In case of repetitive killing assays, residual tumor spheroid fragments were taken out of each well by manual pipetting.

### Animal Experiments

For xenograft mouse models, we tested PA-1 tumor cells, however those cells did not form defined solid but rather diffuse tumors. Instead, we used NCI-N87^CLDN18.2+^ or CT26^CLDN18.2+^ cells. A total of 5 × 10^6^ NCI-N87^CLDN18.2+^ or 5 × 10^5^ CT26^CLDN18.2+^ cells were subcutaneously injected in 100 μL PBS into right back flank of mice. Tumor sizes were measured with a caliper every 2 to 3 days for calculating tumor volumes using the equation (width^2^ × length)/2. Animals were randomly allocated to treatment cohorts using the excel plug-in “Daniel's XL Toolbox” (37). Tumor mean of experimental cohorts is shown with last observation carried forward until over 50% of animals reached experimental endpoint. Data sampling of objective criteria was performed nonblinded.

Retrovirally transduced T cells were adoptively transferred in 200 μL PBS via injection into the retrobulbar venous plexus. For syngeneic mouse experiments, total body irradiated mice were used [XRAD320 device; Precision X-Ray (38)]: C57BL/6BrdCrHsd-*Tyr^c^*, 2.5 Gy; BALB/c, 4 Gy. Irradiations were performed as indicated either 7 days or 1 day prior ACT using XRAD320 device (Precision X-Ray). For NSG mouse experiments, frozen CAR T cells were thawed, washed with PBS and adoptively transferred. For CAR T-cell *in vivo* expansion studies, CAR-Luc-GFP reporter constructs were transduced. CAR T cells without reporter gene coexpression were applied to study antitumor efficacy with or without RNA-LPX combination therapy. To test for persistence and phenotype of transferred CAR T cells *in vivo*, blood was drawn from vena fascialis for flow cytometric analysis. For syngeneic mouse experiments, Thy1.1 was used to distinguish transferred from endogenous T-cell populations. To determine the memory phenotype of transferred T cells, Thy1.1^+^CD8^+^ T cells were assessed for their KLRG1 positivity. KLRG1^neg^ murine T cells were subgated by CD127 and CD62L. KLRG1^neg^CD127^+^CD62L^+^ were determined as central memory T cells, while KLRG1^neg^CD127^+^CD62L^neg^ cells characterized effector memory T cells. In xenograft experiments, adoptively transferred human T cells were identified via hCD45 staining. For RNA vaccination studies, RNA-LPX containing 20 μg antigen-encoding RNA were produced as described previously (25). To test RNA-mediated expansion d-luciferin was administered intraperitoneally into mice. After 5 minutes, bioluminescence was measured via an IVIS Spectrum *in vivo* Imaging System (PerkinElmer). The antitumor function of CLDN18.2-specific TCAR in xenograft, syngeneic *in vivo* expansion and tumor experiments were tested once. For detailed description of *in vivo* experiments, please refer to Supplementary Materials and Methods.

### Statistical Analysis

Descriptive statistics of flow cytometry data was performed using FlowJo v10.6.2. Inferential statistical analysis of datasets was performed using GraphPad Prism 6 or 9. All results are represented with mean + SD of technical replicates or + SEM of biological replicates. Unpaired *t* test was used to compare for statistical significance between slope of linear regressions. Two-way ANOVA tests were performed in case of comparison of experimental data of two or more groups with either one or two independent variables. Multiple comparison testing was done using the Sidak test. Analysis of mice with tumors <1,500 mm^3^ was performed with Mantel-Cox. *, *P* ≤ 0.05; **, *P* ≤ 0.01; ***, *P* ≤ 0.001; ****, *P* ≤ 0.0001.

### Data and Materials Availability

The CLDN6 and CLDN18.2-specific antibodies IMAB027 and IMAB362 and their respective idiotype specific antibodies are courtesy of Astellas Pharma GmbH. Material requests should be addressed to Astellas Pharma GmbH. The data generated in this study are available upon request from the corresponding author.

## Results

### A TCAR Format Mediates Improved Chain Pairing and CD3 Recruitment

**FIGURE 1:**
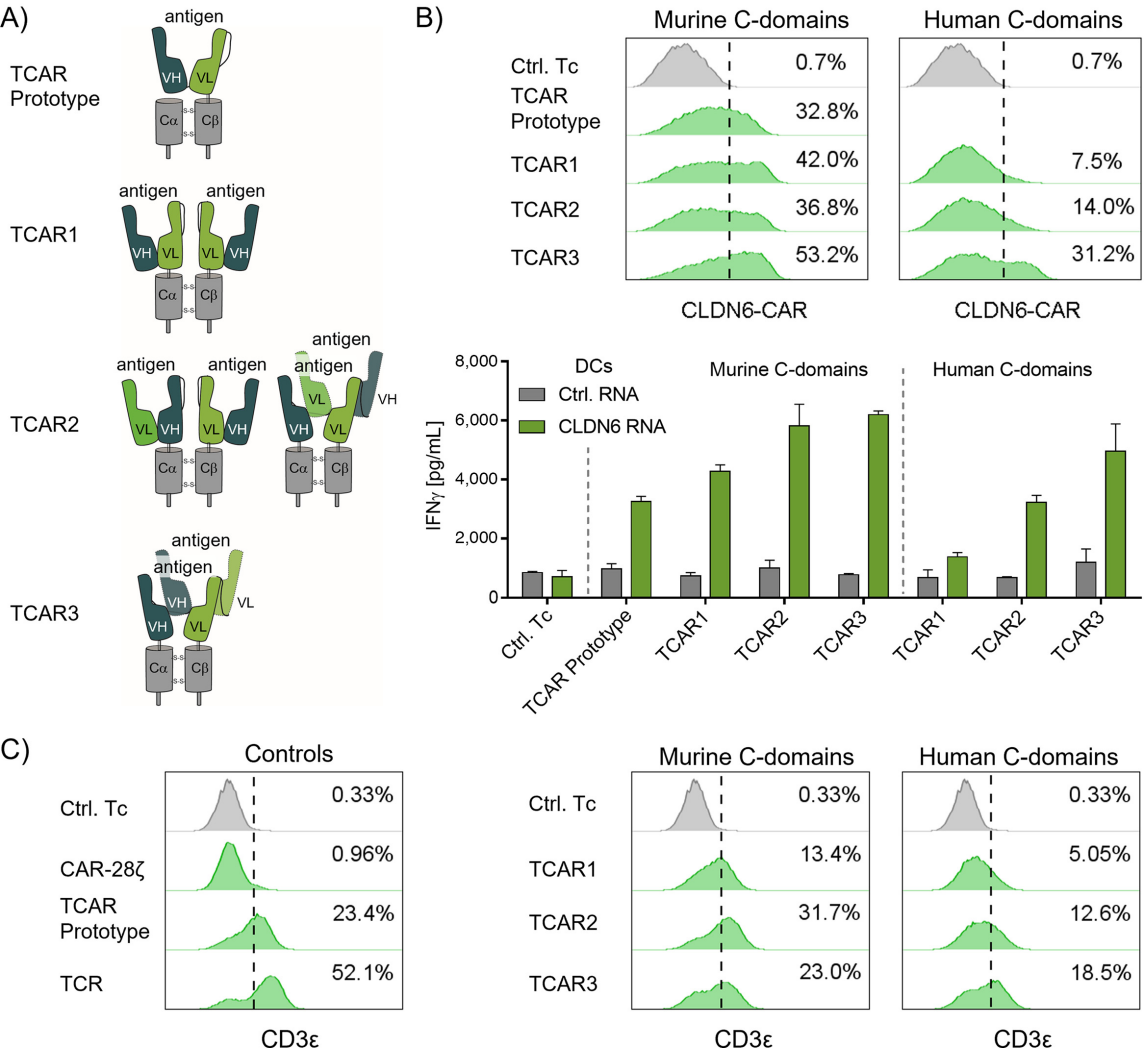
Development of a TCR-like CAR format (TCAR) that mediates antigen recognition and CD3 recruitment. **A,** Schematics of four TCAR formats designed by fusing CLDN6-specific antibody variable domains VH and VL in different orientations to either mouse or human TCR constant domains. The membrane-proximal native and the distal artificial disulfide bonds are indicated. **B,** Surface expression (top) and antigen-specific stimulation (bottom) of different TCARs after RNA transfer in CD8^+^ T cells. TCAR surface expression was assessed by flow cytometry. Cells were gated on control CD8^+^ T cells and dashed line indicates threshold for gating of CAR^+^/CD8^+^ T cells. Antigen-specific stimulation of TCAR T cells was evaluated by IFNγ ELISA (E:T = 10:1) after 20 hours of coculture with CLDN6^+^ DCs. T cells transfected with prototype β-chain RNA only (Ctrl.Tc) and DCs transfected with gp100 RNA (Ctrl. RNA) were used as controls. Graph shows mean + SD of technical duplicates. **C,** Surface expression of CD3ε with different TCAR formats, tested in Jurkat76 cells, which lack endogenous TCR and therefore surface expression of CD3. Murine CLDN6-TCR (TCR), CLDN6-CAR-28ζ, and TCAR prototype α-chain only (Ctrl.Tc) were used as controls.

We initially set out to develop a TCAR by testing four alternative formats (TCAR prototype, TCAR1, TCAR2, and TCAR3; Fig. 1A). They were developed with murine or human TCR α/β constant domains (Cα and Cβ) and CLDN6-specific variable domains (VH and VL). The TCAR prototype construct was generated by fusing the scFv to the Cβ domain and was coexpressed with an autonomous Cα molecule. The TCAR1 and TCAR2 formats had an additional scFv domain fused to the TCR Cα domain in either the same [TCAR1: VH-VL-Cα/ VH-VL-Cβ (2)] or inverse (TCAR2: VL-VH-Cα/ VH-VL-Cβ) orientation to the counterpart, respectively. For TCAR3, the VH and VL domains were separated and arranged in tandem on the TCR Cα/Cβ-chains (VH-VH-Cα/VL-VL-Cβ). While antigen binding by TCAR3 should require engagement of variable domains from both chains, TCAR1 should bind the antigen within a scFv on one chain and TCAR2 should accommodate both types of antigen interaction. To allow strong chain pairing, expression and function of the different TCAR formats, an artificial disulfide bond was integrated between the constant domains of each receptor [Fig. 1A (26)].

To test the function of each of the TCAR formats, they were transfected into human T cells and cultured together with CLDN6-transfected autologous DCs (CLDN6^+^ DC), and IFNγ secretion was measured as an indicator of antigen recognition. The TCAR prototype, by design, was cotransfected with a mouse Cα domain to achieve heterodimerization and integration into the CD3 signaling complex. All four TCAR formats with mouse constant domains were found to be robustly expressed on the T-cell surface and showed antigen-specific recognition of target cells (Fig. 1B). The TCAR prototype was the least effective of the four formats at inducing IFNγ secretion, and also did not induce T-cell proliferation against CLDN6^+^ DCs (Supplementary Fig. S1), potentially due to low receptor-mediated signaling. TCAR2 and particularly TCAR3, both capable of binding the antigen between both chains, were superior to both the TCAR prototype and TCAR1 regarding surface expression and IFNγ secretion (Fig. 1B).

Mouse TCR constant domains were initially chosen for the construct because they stabilize TCR dimer formation (39), but they may induce human anti-mouse antibodies when used clinically (40). We therefore also tested the TCAR formats 1–3 with human C-domains. All CARs were expressed on the cell surface of T cells. While TCAR1 and 2 were characterized by low surface expression and antigen-specific IFNγ secretion (Fig. 1B, right), TCAR3 was the format with most robust expression and mediated prominent IFNγ secretion.

To assess the impact of chain interaction and valency on the activity of TCAR3, we generated and tested three sequence variants of this construct. Backmutation of the artificial disulfide-bond integrated between the human constant domains, which reduces interaction between them (26) consequently reduced TCAR3 function (Supplementary Fig. S2A). Importantly, truncation of the N-terminal VH and VL domains on both TCAR3 chains, reduced the interaction between the two receptor chains, which almost completely abolished its function (Supplementary Fig. S2B). Mutation of the distal VH1 domain (mutVH1), which affects antigen-binding avidity, resulted in strongly impaired TCAR3 functionality, while mutations in the proximal VH2 domain (mutVH2) did not (Supplementary Fig. S2C). Taken together, these results indicate that tightness of interaction between the receptor chains, rather than avidity, improves function of the TCAR3 and allows for human TCR constant domains in its scaffold.

Interaction between TCR constant domains strongly impacts assembly with the CD3 signaling complex and subsequent downstream signaling. We therefore evaluated CD3 recruitment by TCAR1–3 by assessing surface expression of the subunit CD3ε. We used the T-cell leukemia cell line Jurkat76 (J76) that lacks endogenous TCR expression and hence CD3 surface translocation (41). TCAR-positive J76 cells showed surface expression of the endogenous CD3 subunit, CD3ε (Fig. 1C, left). As expected, we observed CD3ε translocation for the TCAR prototype, but not for CAR-28ζ. The TCAR1–3 with mouse C-domains recruited CD3ε to the cell surface more efficiently than TCAR1–3 with human C-domains (Fig. 1C, middle and right). Among the TCAR formats with human C-domains, TCAR3 led to the highest CD3ε signals (Fig. 1C, right). Because CD3ε forms heterodimers with CD3δ and CD3γ, CD3ε surface expression served as proxy for full CD3 complex recruitment. Co-immunoprecipitation studies with J76 cells confirmed that TCAR3 associated with CD3ζ and CD3γ chains, while CAR-BBζ did not (Supplementary Fig. S3). On the basis of these aggregate data, TCAR3 with human constant domains, from now on referred to as TCAR, was selected for further characterization studies in human T cells.

### TCAR Mediates Highly Sensitive Antigen Recognition

**FIGURE 2:**
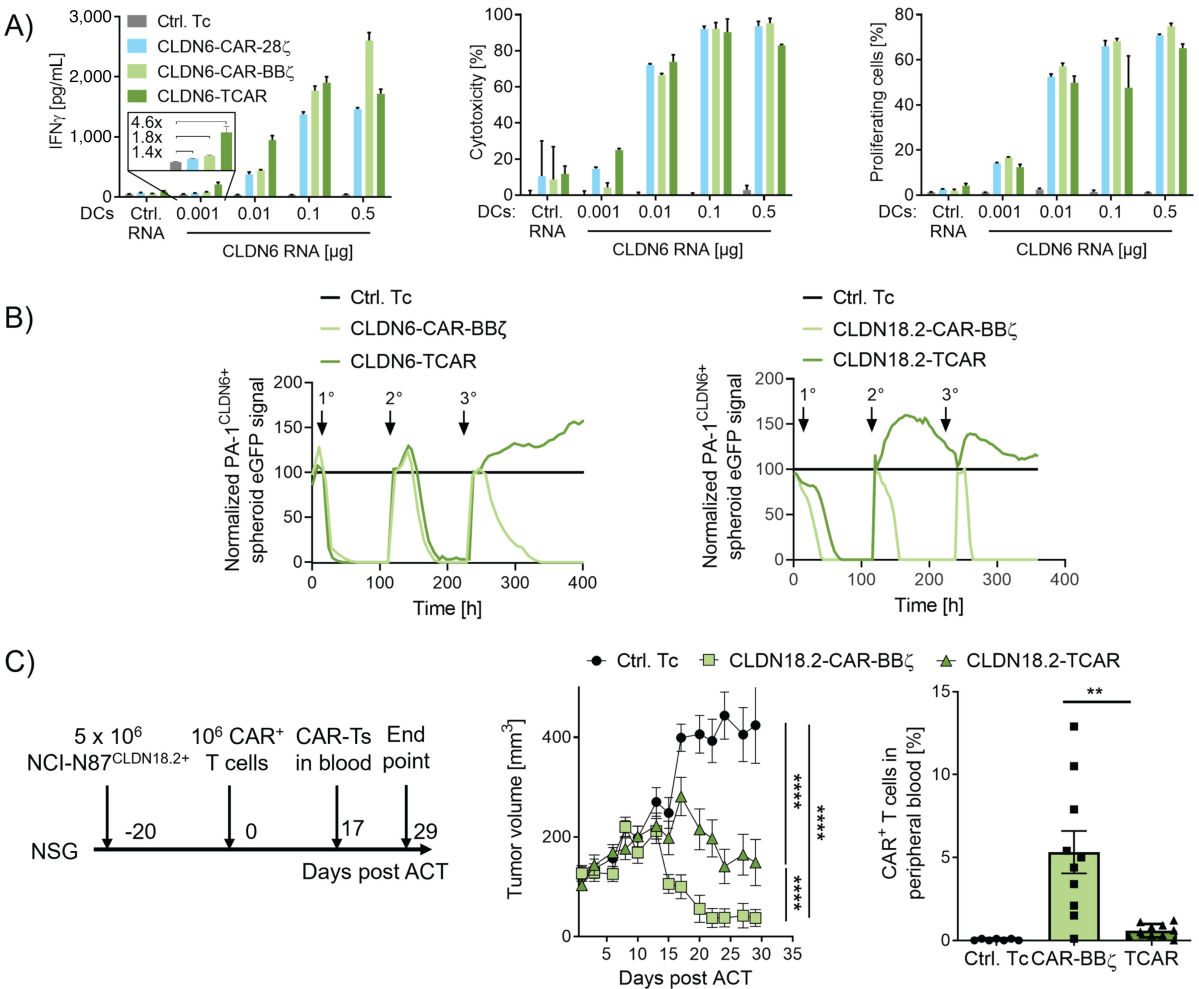
TCAR mediates highly sensitive antigen recognition and antitumor function. **A,** IFNγ secretion (left), cytotoxicity (middle), and proliferation (right) of CLDN6-TCAR– or CLDN6-CAR–expressing human CD8^+^ T cells cocultured with CLDN6^+^ DCs [E:T ratio = 10:1 (left and middle) or 20:1 (right)]. IFNγ concentrations in culture supernatants were determined after 20 hours of coculture by IFNγ ELISA. Cytotoxicity and proliferation of CLDN6-TCAR and CAR T cells was assessed by luciferase-based cytotoxicity and CFSE proliferation assays. T cells transfected with Cα RNA only (Ctrl. Tc), and DCs transfected with CLDN18.2 RNA (Ctrl. RNA) were used as negative controls. Graphs show mean + SD of technical duplicates (left, right) or triplicates (middle) representative for 3 blood donors. **B,** Serial killing of human CLDN6^+^ or CLDN18.2^+^ PA-1 tumor spheroids by CLDN6- (left) or CLDN18.2- (right) TCAR/CAR-transduced CD3^+^ T cells analyzed by eGFP real-time imaging (E:T = 30:1). Nontransduced cells (Ctrl. Tc, left) or CD19-CAR-BBζ transduced T cells (Ctrl. Tc, right) were used as controls. Arrows indicate addition of tumor spheroids. Mean of technical triplicates is shown. **C,** NSG mice with subcutaneous NCI-N87^CLDN18.2+^ xenografts were treated with human TCAR/CAR-transduced T cells (10^6^ cells/animal, 10 mice/group) or nontransduced T cells (Ctrl. Tc; 10 mice/group; left) at a mean volume of 98 mm^3^. Tumor growth kinetics (middle) and TCAR^+^/CAR^+^ T-cell frequency in peripheral blood 17 days post ACT (right) as analyzed by flow cytometry. Graphs show mean + SEM of biological replicates. Two-way ANOVA analysis with Sidak posttest were used to test for statistical significance. **, *P* ≤ 0.01; ****, *P* ≤ 0.0001.

Next, we characterized TCAR function in RNA-transfected human T cells *in vitro*. In cocultures with CLDN6^+^ DCs, the extent of T-cell responses was largely comparable with that of second-generation CLDN6-CARs and depended on the CLDN6 expression level on DCs (Fig. 2A). CLDN6-TCAR T cells exerted robust IFNγ secretion, cytotoxicity, and proliferation. Notably, in IFNγ secretion assays, TCAR T cells recognized even ultralow antigen densities on CLDN6^+^ DCs (Fig. 2A, left). We observed that in human T cells transfected with high but equimolar amounts of receptor-encoding RNA, surface expression of CLDN6-TCAR was lower than that of second-generation CARs (Supplementary Fig. S4A). This disparity in surface expression increased with escalating amounts of transfected receptor-encoding RNA (Supplementary Fig. S4B).

Because TCAR surface expression is reliant on CD3 recruitment and CAR-BBζ is not, this result suggested that the availability of CD3 subunits and competition for them with endogenous TCR molecules may be a limiting factor in surface expression of TCAR (42). We therefore assessed the relationship between receptor surface expression and IFNγ secretion upon recognition of CLDN6^+^ DCs. For any given receptor expression level, the IFNγ secretion response was higher with CLDN6-TCAR than CLDN6-CAR-BBζ T cells suggesting a higher sensitivity of CLDN6-TCAR T cells (Supplementary Fig. S4C). We confirmed this assumption by testing cytotoxicity against the human SK-OV-3 ovarian cancer cell line, which expresses CLDN6 endogenously at extremely low levels (Supplementary Fig. S4D and S4E).

Because differences in receptor expression induced strong variation in T-cell function, for all following T-cell assays the percentage of CAR T cells was calibrated (unless otherwise indicated) to ensure comparability between receptor types.

For early proof-of-concept studies, we used transient expression of receptor-encoding RNA in T cells. To validate TCAR function in long-term experiments better reflecting the *in vivo* setting, we retrovirally transduced T cells for stable antigen receptor expression. We used titrated retroviral supernatants and an multiplicity of infection of 1 for transductions to achieve a maximum of one copy per cell, which corresponds to approximately 30% of CAR-expressing T cells. Both CLDN6- and CLDN18.2-specific TCAR T cells generated by viral transduction showed marked surface expression and sensitive recognition and killing of CLDN6^+^ and CLDN18.2^+^ DCs (Supplementary Fig. S5).

### Costimulation Promotes TCAR Function and Persistence

Next, we conducted a series of experiments to assess the performance of TCAR T cells upon engagement with human tumor cell lines. CLDN6-TCAR T cells exerted potent and antigen-dependent IFNγ secretion and cytotoxicity against human CLDN6^+^ Colo-699-N lung cancer cells (Supplementary Fig. S6A and S6B, left and middle). However, proliferation of these cells was low, particularly in comparison with proliferation of CLDN6-CAR-BBζ T cells (Supplementary Fig. S6B, right).

In a repetitive *in vitro* killing assay, CLDN6- or CLDN18.2-TCAR and CAR-BBζ T cells were challenged three times at 5- to 7-day intervals with tumor spheroids derived from CLDN6^+^ or CLDN18^+^ PA-1 cells (Fig. 2B). The repetitive spheroid assay was performed in the absence of exogenous cytokines thereby restricting CAR- and TCAR-T cell function to autocrine and paracrine signaling. It requires potent T-cell killing, T-cell survival, and proliferation over multiple rounds of antigen exposure and represents a challenging *in vitro* test system. At first encounter, both CLDN-TCAR T-cell types effectively cleared tumor spheroids. They failed to do so at subsequent spheroid challenges, while CLDN-CAR-BBζ T cells could resolve all tumor spheroids after all three encounters. TCAR-T exhaustion and memory phenotypes could not be determined because of low residual TCAR T-cell numbers in the cultures indicating that missing TCAR T-cell survival and enrichment was the cause for spheroid outgrowth.

Next, we assessed TCAR-mediated antitumor activity *in vivo* using a xenograft mouse model. Mice with large human tumors derived from CLDN18.2^+^ NCI-N87 cells (mean volume 98 mm^3^) underwent ACT with a single dose of human CLDN18.2-TCAR T cells, CLDN18.2-CAR-BBζ T cells, or nontransduced control cells, respectively. While tumors in the control group progressed, treatment with receptor engineered T cells reduced tumor growth (Fig. 2C). However, CLDN18.2-TCAR T cells were not as efficient as CLDN18.2-CAR-BBζ T cells, which mediated complete tumor regression in all mice within 4 weeks (Fig. 2C, middle).

**FIGURE 3:**
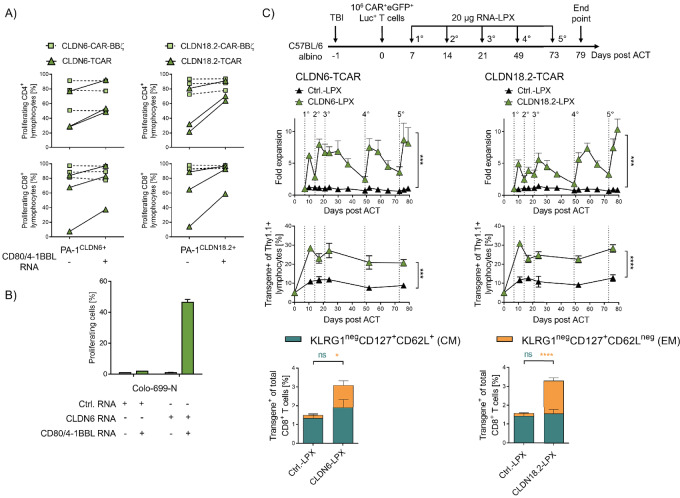
Costimulation promotes TCAR function and expansion *in vitro* and *in vivo*. **A** and **B,** Expression of costimulatory molecules on tumor cells increases proliferation of human CLDN6- and CLDN18.2-specific TCAR T cells after coculture. **A,** The proportion of proliferating transduced T cells after coculture with CLDN6^+^ or CLDN18.2^+^ PA-1 cells with or without transfection of costimulatory molecules CD80 and 4-1BBL (E:T 10:1). Mean + SD of technical duplicates of three blood donors are presented. **B,** The proportion of proliferating receptor-transfected T cells after coculture with CLDN6^+^ Colo-699-N cells (E:T = 3:1) with or without costimulatory molecules CD80 and 4-1BBL. Control, CLDN9-transfected Colo-699-N cells (Ctrl. RNA). Mean + SD of technical duplicates. **C,** RNA-LPX vaccination mediates TCAR T-cell expansion in syngeneic *in vivo* mouse model. Luc-GFP–expressing Thy1.1^+^ CLDN6- or CLDN18.2-TCAR mouse T cells were transferred into total body irradiated (TBI) Thy1.2^+^ C57BL/6-albino mice (*n* = 5 mice/group). Seven days later, mice were injected with 20 μg CLDN6 or CLDN18.2 RNA-LPX. Kinetics of TCAR T-cell expansion by bioluminescence imaging (BLI, top) and the frequencies of TCAR^+^/eGFP^+^/Thy1.1^+^ lymphocytes in peripheral blood (middle) are shown. Frequency of effector memory (EM) and central memory (CM) TCAR T cells in the CD8^+^ T-cell population 52 days after ACT is shown (bottom). CLDN18.2-LPX served as negative control (Ctrl. LPX) for CLDN6-TCARs, and CLDN6-LPX as a negative control for CLDN18.2-TCARs. Mean + SEM of 5 mice per group is shown. Two-way ANOVA analysis with Sidak posttest were used to test for statistical significance. ns, not significant; *, *P* ≤ 0.05; **, *P* ≤ 0.01; ***, *P* ≤ 0.001; ****, *P* ≤ 0.0001.

Frequency of adoptively transferred CLDN18.2-TCAR T cells in peripheral blood of these mice was found to be low 17 days after adoptive T-cell transfer (Fig. 2C, right). As persistence of transferred CAR T cells is known to be critical for their clinical effect (43–45), we looked for ways to increase the persistence of the TCAR T cells. One prerequisite for strong T-cell proliferation and survival is the presence of costimulatory signals, inherent to DCs used in our earlier experiments, but lacking in tumor cells. By transfecting CLDN^+^ PA-1 and Colo-699-N tumor cells with costimulatory molecules CD80 and 4-1BBL (ref. 46; Supplementary Fig. S7A), we markedly improved antigen-dependent proliferation of CLDN-TCAR transfected or transduced T cells. CLDN-CAR-BBζ T cells did not benefit further from this provision of costimulatory signals, presumably due to costimulation already provided by the CAR construct itself (Fig. 3A and B)*.*

We next verified the effect of costimulation in the repetitive *in vitro* killing assay. CLDN-TCAR T cells rechallenged with spheroids coexpressing costimulatory molecules cleared CLDN^+^ spheroids more successfully than those challenged without costimulation (Supplementary Fig. S7B). Similarly, rechallenge with CLDN^+^ DCs as a physiologically relevant source of costimulation, improved the efficiency of TCAR T cells at killing CLDN^+^ PA-1 spheroids (Supplementary Fig. S7C).

### *In Vivo* Salvage via CARVac RNA-LPX Vaccination Promotes TCAR Activity Against Solid Tumors

Having shown that for sustained functionality and proliferation, TCAR T cells benefit from costimulation along with target antigen engagement *in vitro*, we explored *in vivo* translation of this concept. We have previously reported a nanoparticulate RNA-LPX vaccine (25), which delivers the CAR antigen bodywide into lymphoid compartments for antigen-specific stimulation of the transferred CAR T cells. Presentation of the natively folded target antigen on resident DCs in the lymphoid tissue was accompanied with activation of the DCs and thus local augmentation of costimulatory signals (20). We evaluated the upregulation of costimulatory molecules after RNA-LPX treatment and demonstrated antigen-specific expansion of CLDN6- and CLDN18.2-CAR-BBζ T cells *in vivo*. Improved engraftment of CAR T cells and regression of large tumors in mouse models was achieved at subtherapeutic CAR T-cell numbers (20).

We further asked whether this so-called CARVac approach could be used to provide costimulation and thus enhance the performance of TCAR T cells *in vivo*. First, we treated DCs with titrated amounts of CLDN6-encoding RNA-LPX. The resulting expression of CLDN6 on DCs induced stimulation, cytokine secretion, and proliferation of cocultured CLDN6-TCAR T cells in a dose-dependent manner (Supplementary Fig. S8).

Next, we evaluated the CARVac approach *in vivo*. Because of the suboptimal lymphoid compartments in NSG mice, we switched to syngeneic mouse models. We first confirmed the upregulation of costimulatory molecules on splenic DCs after RNA-LPX treatment (Supplementary Fig. S9A). Thy1.2^+^ C57BL/6 mice underwent total body irradiation and were engrafted with 10^6^ congenic (Thy1.1^+^) mouse T cells transduced with CLDN6- or CLDN18.2-TCAR (containing mouse C-domains) coexpressing luciferase and eGFP for *in vivo* bioluminescence imaging and flow cytometric analysis (VL-VL-mCβ-P2A-VH-VH-mCα-T2A-luc-eGFP). We used TCARs with murine C-domains as surrogates in murine T cells to circumvent the weak competition of TCARs carrying human C-domains with the endogenous mouse TCRs for CD3 recruitment. Starting 7 days after ACT, mice were vaccinated weekly for 21 days with 20 μg RNA-LPX encoding CLDN6 or CLDN18.2. Bioluminescence imaging revealed that a single vaccination of RNA-LPX induced rapid expansion of TCAR T cells (Fig. 3C, top; Supplementary Fig. S9B). This expansion of TCAR T cells peaked 3–4 days after vaccination and was followed by a subsequent contraction phase, mimicking the physiologic T-cell response. Consecutive RNA-LPX administrations in the following weeks induced similar kinetics. Importantly, no *in vivo* expansion of CLDN6- or CLDN18.2-specific TCAR T cells was observed when RNA-LPX encoding a control antigen was administered. Antigen-specific activation of CLDN6- or CLDN18.2-specific TCAR T cells showed comparable expansion rates. As expected, CLDN-specific CAR-BBζ-T cells expanded strongly upon RNA-LPX treatment due to their in-build costimulatory signaling moieties (Supplementary Fig. S10). After initial expansion, mice were vaccinated again after a 28- and then 24-day treatment break to examine the expansion potential of TCAR T cells at later timepoints after treatment breaks and assess exhaustion of the T cells after repetitive stimulation. Indeed, a large increase in TCAR T-cell frequency was achieved, as indicated by luciferase signals reaching even higher levels than after initial expansion. Flow cytometric analysis of peripheral blood confirmed stable engraftment and persistence of TCAR T cells supported by RNA-LPX administration (Fig. 3C, middle). Accordingly, assessment of T-cell phenotype revealed that antigen-specific RNA-LPX vaccination induced an enrichment for effector memory-like TCAR T cells (Fig. 3C, bottom).

**FIGURE 4:**
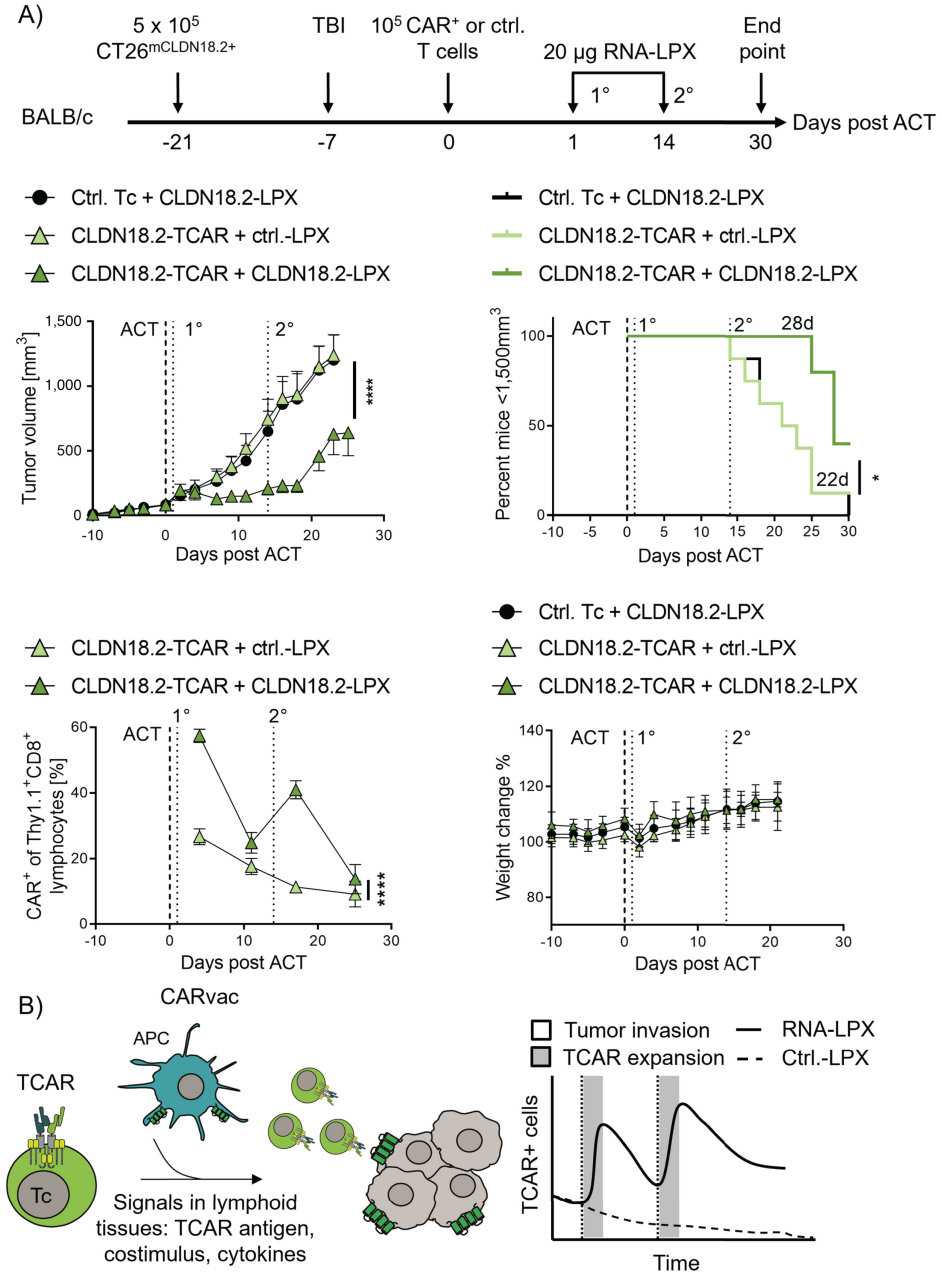
CARVac promotes TCAR-T *in vivo* expansion and efficacy against solid tumors in syngeneic *in vivo* mouse model. **A,** BALB/c mice bearing CLDN18.2^+^ CT26 approximately 87 mm^3^ tumors (*n* = 10 mice per group) were lymphodepleted by total body irradiation (TBI) and treated with syngeneic CLDN18.2-TCAR mouse T cells (5 × 10^5^ cells/animal) followed by two intravenous administrations of 20 μg CLDN18.2 RNA-LPX. eGFP-transduced T cells (Ctrl. Tc) and 20 μg CLDN6 RNA-LPX (Ctrl. LPX) were used as negative controls. **A**, top left, Tumor growth kinetics is shown as mean + SEM tumor volume. ****, *P* ≤ 0.0001 by two-way ANOVA analysis with Sidak posttest. **A**, top right, Percentage of mice with tumor mass below 1,500 mm^3^ is shown. Numbers indicate median time until tumors reached 1,500 mm^3^. ****, *P* ≤ 0.0001 by Mantel–Cox test. **A**, bottom left, TCAR frequency based on proportion of Thy1.1^+^ CD8^+^ T cells in blood determined by flow cytometry. Mean + SEM is shown. *, *P* < 0.05 by two-way ANOVA analysis with Sidak posttest. **A**, bottom right, Percent of initial mouse body weight. **B,** Idealized study summary: after specific binding to its target antigen on the surface of APCs, the TCAR initiates TCR-like signals for potent T-cell activation and effector function. RNA-LPX mediated provision of costimulation and cytokine support via dendritic cells in lymphoid tissues expands TCAR T cells, leads to increased TCAR T cells in the blood, contraction, and tumor infiltration for an improved antitumor efficacy. Ctrl.-LPX encoding an TCAR irrelevant antigen.

To assess the impact on antitumor activity, BALB/c mice with large mouse CLDN18.2^+^ CT26 colon tumors (mean tumor volume 87 mm^3^) were submitted to adoptive cell transfer with a subtherapeutic dose of mouse Thy1.1^+^ CLDN18.2-TCAR T cells followed by two injections (2 weeks apart) of CLDN18.2-LPX or control LPX (Fig. 4A, top row). While CLDN18.2-TCAR T cells alone had no effect on tumor growth, combining them with vaccination significantly delayed tumor growth and prolonged survival (Fig. 4A, middle row). Each of the vaccine doses was found to increase TCAR-expressing T cells in peripheral blood followed by a contraction of this adoptively transferred T-cell population (Fig. 4A, bottom left). No signs of toxicity were observed in any of the treatment groups by visual inspection and the body weight of all animals remained stable (Fig. 4A, bottom right).

## Discussion

CAR designs have greatly evolved in the last three decades, which has resulted in the approval of CAR therapies for hematologic disorders. However, clinical success of these therapies for solid malignancies is so far limited. In this article, we show a novel TCAR design with potent effector functions and higher sensitivity than second-generation CARs. In contrast to the latter, TCAR does not contain costimulatory signaling moieties like 4-1BB or CD28. Consequently, we observed lower antigen-specific expansion of TCAR T cells than second-generation CARs against tumor cells *in vitro* and *in vivo*. However, by providing antigen-specific costimulation via DCs or tumor cells modified with costimulatory molecules, TCAR T cells exhibited robust proliferation *in vitro*. Similarly, application of the CARVac technology to provide costimulation *in situ* through endogenous DCs residing in lymphatic organs promoted TCAR T-cell expansion *in vivo*. The expansion phase was followed by a contraction phase of the TCAR-T population. Accordingly, coadministration of TCAR T cells together with CARVac increased antitumor activity. An idealized study summary is depicted in Fig. 4B.

For physiologic T-cell activation, three signals are required. First, binding of the TCR to pMHC molecules on the surface of APCs leads to phosphorylation events on the adjacent CD3 complexes, initializing a signaling cascade responsible for proximal T-cell activation. Second, interaction of costimulatory ligands such as CD80 and 4-1BBL on APCs with CD28 and 4-1BB receptors on the surface of T cells supports this T-cell activation. Third, additional cytokines support and tune the T-cell activation and differentiation. In the case of second-generation and third-generation CARs, both TCR and costimulatory signals are provided by the CAR scaffold itself. We, however, wanted to address the question of how a TCAR scaffold would perform, and designed a TCAR that retargets T cells toward a surface antigen using the endogenous T-cell CD3 signaling complexes for physiologic TCR-like activation.

The TCAR contains an innovative tandem configuration of variable domains (VH1-VH2-Cα and VL1-VL2-Cβ) that sets it apart from the single-chain arrangements found in other candidates that we and others tested, and from previous second-generation CAR formats. In TCAR, the antigen can serve as a “noncovalent linker” across both receptor chains as reported for soluble monomeric and dimeric scFvs (47). This, in combination with the tandem configuration of V-domains, might result in higher α/β-chain pairing of the TCAR along the entire polypeptide chains, thus strengthening the TCAR/CD3-complex. Truncation of the N-terminal VH1 and VL1 domains in the respective chains, a structure similar to the recently published STAR receptor (48), completely abolished function of a TCAR with human C-domains (Supplementary Fig. S2B). However, mutation of the proximal VH2 had only a minor effect. This demonstrated that TCAR function is less determined by its valency, than by its strong chain interaction. Notably, and in contrast to the STAR receptor format, this allows for incorporation of human TCR C-domains in the TCAR (Supplementary Fig. S2). In TCRs, an increase in interaction between α- and β-chains is associated with a higher TCR/CD3 interaction (39), which also underlies the observed variations in CD3 recruitment by different TCARs (Fig. 1). However, it is well known that human TCR α/β-domains do not have adequate domain pairing forces in mouse T cells to recruit endogenous CD3 for surface expression (49). Hence, we circumvented the weak expression of TCARs in mouse T cells by taking TCARs with murine Cα/β domains as a surrogate for *in vivo* experiments. Although murine Cα/β TCARs exhibited improved expression and function *in vitro* (Fig. 1) and pronounced antitumor reactivity in mice, we do not propose using them in future clinical trials but suggest using human Cα/β TCARs. With the latter, lower immunogenicity would be expected and hence, long-term persistence of TCAR-T *in vivo*.

By incorporation into the natural CD3 complex, and without fusions to costimulatory moieties, the TCAR follows a different and more physiologic T-cell activation than previous CAR designs and induces strong and sensitized responses against its antigen *in vitro* and *in vivo*. Davenport and colleagues observed the formation of nonclassical immune synapses characterized by multifocal Lck clusters in CAR-T as opposed to the monocentric synapse formation in TCR-T cells (11). This in turn resulted in different T-cell signaling kinetics. Recently, Gudipati and colleagues reported on reduced antigen sensitivity of CAR-T due to inefficient recruitment of the most proximal adaptor protein ZAP-70 in T-cell signaling (12). These alterations in CAR-T-cell signaling most likely result from a different signalosome formation (TCR/CD3 vs. CAR clustering) in the immune synapses upon antigen encounter eventually impacting their effector function.

One proposed factor underlying those observations in second-generation CARs could be the absence of CD3ε ITAMs in their design. The latter have been shown to influence Nck recruitment, actin dynamics, maturation, and function of the immune synapses (50–52). Incorporation of CD3ε together with CD3ζ into a CAR-28ζ has consequently been shown to improve persistence of CAR T cells *in vivo* (53). But even incorporation of CD3ε into a CAR scaffold might not solve issues like tonic signaling seen for some second-generation CARs. Here, we postulate that TCARs in contrast to second-generation CARs form aggregates with CD3 chains highly related to antigen-sensitive TCR/CD3 complexes.

The intrinsic sensitivity of the TCAR could be used for the treatment of antigen-low solid tumor entities or hematologic malignancies such as CD19 leukemia or lymphomas to prevent relapse of B cell–derived tumor cells (54), the latter even providing costimulation in trans. Although we have observed a major contribution of the distal VH1 to antigen binding in the antigen monospecific TCAR, a bispecific TCAR may further decrease the chance of antigen escape variants in hematologic or solid tumors.

Other approaches that exploit the endogenous CD3 complex for signaling have been published previously (55–57). T cell membrane–bound bispecific antibodies targeting CD3 or tumor antigens (TAC) or alternatively scFv-CD3 fusions (TRuC) have been used to cluster the endogenous TCR/CD3 complexes. But in contrast to the TCAR design, they could not benefit from endogenous TCR knockout strategies (42, 58–60). This intervention likely reduces alloreactivity for off-the-shelf approaches and increases TCAR function. However, TCR knockouts can also improve safety of a T-cell product by reducing off-target toxicities mediated by transactivation of the endogenous TCR (61). In addition, CARs with fusions of Fab fragments to the constant domains of the γδTCR also recruit the T-cell endogenous CD3 complexes and show function in hematologic tumor models (57). However, γδTCRs reveal a quaternary structure varying from αβTCRs and expose different surfaces to CD3 subunits (62). Consequently, γδTCR constant domains are considered to show lower recruitment of CD3δε, implying distinct signal routes and strengths (63). This might interfere with the meticulously regulated activation and negative feedback loops seen in αβ-T cells. Of note, all previously mentioned CD3 complex–dependent CAR scaffolds were argued to induce lower toxicity due to lower cytokine secretion. However, we observed a reduction in cytokine secretion for TCAR T cells only upon exposure to high amounts of target antigen, pointing toward a negative feedback mechanism of this novel receptor (Fig. 2A and Supplementary Fig. S6, left). In addition to the previous constructs, a recent publication by Liu and colleagues (48) provided insight into the function of the STAR receptor, a CAR (VH-mCα + VL-mCβ) similar to the truncated version of TCAR (VH-hCα + VL-hCβ) presented in this article, but with murine C-domains. In line with our observations (Supplementary Fig. S2), the STAR receptor elicited strong effector functions, such as cytokine secretion. However, contradictory to our data and other published studies (64–66), this receptor did not require costimulation for proliferation induction against tumor cells and could even outperform second-generation CARs (48).

Our novel TCAR design represents an alternative receptor scaffold to induce potent T-cell activity against tumor cells also in the context of solid malignancies. MHC-independent and well-regulated specific T-cell activation, in combination with the strong proximal CD3 signaling, induced potent and antigen-sensitive effector functions, underlining the potential of this novel receptor scaffold. The fact that TCAR T cells did not require fusions of costimulatory signaling moieties for long-term persistence *in vivo* (Fig. 3), supports the notion that this novel receptor construct largely benefits from T-cell activation mediated by the T-cell endogenous signaling machinery. In NSG mouse experiments, we saw potent initial antitumor responses of TCAR T cells (Fig. 2). Compared with T cells carrying the CAR-BBζ with intrinsic costimulation, TCAR T cells showed lower proliferation when exposed to cells not providing costimulation. We argue that this poses a major safety advantage of TCARs over second-generation and similar published CAR approaches, because the lack of costimulus perturbs strong TCAR T-cell expansion and reactivity in healthy tissues which can have fatal consequences due to on-target/off-tumor reactivities (67). Alternatively to the tightly controlled provision of costimulation using CARVac, a recent publication presented the benefit of T cell–encoded CD80 and 4-1BBL on T-cell persistence in the context of an HLA-independent TCR [VH-Cβ + VL-Cα (68)].

The data provided by us and others, demonstrate that CARs recruiting the T-cell endogenous CD3 complexes have high antigen affinity, and benefit greatly from antigen expression and costimulation provided in *trans* via RNA-LPX treatment or even in *cis* via T cell–encoded costimulatory molecules. However, we observed a delayed rather than a complete resolution of tumors *in vivo* in immunocompetent mice. Because RNA-LPX strongly supports *in vivo* expansion of TCAR-T, we argue that their antitumor function could be enhanced by additional mRNA vaccinations. Besides the lack of antigen and costimulation for TCAR-T, the encounter of a strong immunosuppressive tumor microenvironment might also influence the T-cell antitumor function. The more physiologic signaling induced by the TCAR compared with second-generation CARs may render TCAR-T susceptible to coinhibitory signals such as CTLA-4, PD-1, TIM-3, LAG-3 or cytokines like TGFβ and IL10 originating from tumor-associated macrophages, myeloid-derived suppressor cells, regulatory T cells, or even the tumor cells themselves. Genomic modifications of TCAR-T or combination with established therapies like checkpoint inhibitors could abrogate such inhibitory signals. They could, for example, preserve Tcf7-positive T-cell populations characterized by higher capacity for self-renewal (69) and counteract activation-induced cell death (AICD) or exhaustion. The latter state has previously been characterized by upregulation of factors like TOX/TOX2 or members of the NR4A family (70, 71). While coexpression of the TCAR together with the CD3 subunits could increase TCAR-dependent signaling (42) and avoid antigen escape, the previously described TCR knockout strategies combined with TCR locus–targeted TCAR integration could support a fine-tuned TCAR signaling to avoid AICD (60). However, fine tuning of TCAR function alone might not resolve the roadblocks coming from coinhibitory signaling. An increasing number of studies have tested CAR-Ts genetically modified to cope with suppressive signals such as PD-L1 or TGFβ in the TME (72–74). For TCRs, first promising patient data have already been published in this regard (75).

In the clinical setting, our novel TCAR design together with the CARVac strategy could in the future enable physicians to follow an expand-on-demand approach and safely fine-tune TCAR T-cell functions in the patient. The TCAR could become a modular tool to integrate T-cell activating signals together with exogenous TCRs, immunomodulators (76), chimeric costimulatory receptors (77), checkpoint inhibitors, or cytokines to systemically expand genome engineered TCAR-Ts. The T-cell endogenous CD3 subunits recruited by TCAR could help to integrate complex T-cell signaling events and incorporate this biologic in its physiologic T-cell niche.

## Supplementary Material

Supplementary Data and Figures Supplementary Data and Figures
