# Supplementary material for: A TCR-like CAR Promotes Sensitive Antigen Recognition and Controlled T-cell Expansion Upon mRNA Vaccination
Source: Cancer Res Commun. 2022 Aug 18;2(8):827–41. doi: 10.1158/2767-9764.CRC-21-0154 (PMC10010320; doi:10.1158/2767-9764.CRC-21-0154)
Supplement: Supplementary Data and Figures [file crc-21-0154-s01.pdf]

Supplementary Materials and Methods

List of supplementary materials and methods

Cytokine multiplex analysis
RNA-lipoplex (RNA-LPX) generation and treatment
Bioluminescence *in vivo* imaging
Co-immunoprecipitation
Digital droplet PCR analysis of CLDN6 expression in SK-OV-3 cells

References

List of supplementary figures

Fig. S1. CAR-28ζ is more effective than TCAR prototype at inducing IFNγ secretion and proliferation.
Fig. S2. Interaction between variable domains, rather than avidity, is required for TCAR3 function.
Fig. S3. The constant domains of TCAR3 recruit endogenous CD3.
Fig. S4. CLDN6-TCAR surface expression in human $CD8^+$ T cells and recognition of $CLDN6^+$ target cells.
Fig. S5. T cells with stable TCAR expression generated by viral transduction show robust surface expression, and antigen-specific cytotoxicity.
Fig. S6. TCAR T cells mount potent effector functions but lack proliferation against tumor cells.
Fig. S7. Antigen-specific stimulation of CLDN6 and CLDN18.2-CAR T cells via CD80 and 4-1BBL transfected PA-1 spheroids or DCs enhances subsequent tumor spheroid killing.
Fig. S8. CLDN6-TCAR and CAR-BBζ T cells proliferate and secrete cytokines against CLDN6-LPX treated DCs.
Fig. S9. RNA-LPX facilitates upregulation of costimulatory molecules in splenic DCs and expands TCAR-T antigen-specifically.
Fig. S10. RNA-LPX mediated *in vivo* expansion of CAR-BBζ engineered murine T cells.

Cytokine multiplex analysis

The ProcartaPlex™ 3-Plex Kit immunoassay (Invitrogen) with multi-analyte reagent panels for quantitative analysis of human IL-2, IFNγ and TNFα was used to detect cytokines after 24 h in co-cultures using Bio-Plex 2000 according to the manufacturer instructions.

RNA-lipoplex (RNA-LPX) generation and treatment

For RNA-LPX generation, 20 µg antigen-encoding RNA was formulated into liposomes containing a DOTMA to DOPE ratio of 2 to 1 with a net charge ratio of cationic DOTMA to RNA of 1.3 to 2 [25]. For *in vitro* treatment of DCs, cells were seeded in 24 well plates, treated with the respective amounts of RNA-LPX and incubated for 24 h prior to the start of co-cultures. For *in vivo* expansion experiments 200 µL RNA-LPX solutions buffered in 150 mM NaCl were administered into the retrobulbar venous plexus. Negative control lipoplexes were generated with irrelevant RNA mentioned in the respective figure legend. Time points of RNA-LPX treatment are indicated in the relevant graphs.

Bioluminescence *in vivo* imaging

Biodistribution and expansion of CAR-Luc-GFP transduced C57BL/6-Thy1.1$^+$ T cells was evaluated by *in vivo* bioluminescence imaging as previously described [20]. In brief, a solution of D-luciferin (80 mg/kg body weight) was administered intra-peritoneal. Radiance (photon/s/cm^2) was measured 5 minutes later with an exposure time of 1 minute on an IVIS Spectrum *in vivo* Imaging System (PerkinElmer). Prior to T cell expansion, a base line value was obtained. Quantification of CAR T cell expansion are based on total flux of the indicated time point divided by total flux at baseline.

Co-immunoprecipitation

Jurkat76 cells were electroporated with antigen receptors and cultured for 20 h. Cells were pelleted and shock-frozen in liquid nitrogen and stored at -80°C. Pellets of receptor electroporated Jurkat76 cells were resuspended in lysis buffer [20 mM Tris-HCl (pH 8), 137 mM NaCl, 2 mM EDTA, 10% glycerol, 0.3% Brij96V, Halt™ Protease and Phosphatase Inhibitor Cocktail (Thermo Scientific)], incubated on ice for 30 minutes (short mix every 5 minutes) and centrifuged (13,000 ×g, 10 minutes, 4°C) to remove cell debris. 15 µg anti-Myc Tag antibody (Epitope Biotech Inc.) was coupled to 1.5 mg Dynabeads™ Protein G (Thermo Scientific) for 15 minutes at room temperature. The antibody-bead complex was incubated with precleared Jurkat76 cell lysates for 1 h at 4°C on a rotating mixer. Precipitated protein complexes were washed twice with PBS, 0.01% Triton X-100, [pH 7.4], Halt™ Protease and Phosphatase Inhibitor Cocktail and eluted by boiling in 4× SDS sample buffer. Immunoprecipitated protein samples were resolved by 10% C, 3% T Tricine-SDS-PAGE [1] and analyzed by antiCD3γ and CD3ζ immunoblotting (Abcam: ab134096, ab226475). Visualization was done with a CCD camera (ImageQuant LAS4000; GE Healthcare Life Sciences).

Digital droplet PCR analysis of CLDN6 expression in SK-OV-3 cells

Total RNA was extracted from SK-OV-3 and Colo-699-N cell lines using RNeasy Mini Kit (QIAGEN). From fresh frozen placenta TRIzol/chloroform based RNA extraction was performed followed by RNA clean-up using RNeasy Mini Kit (QIAGEN). 1 µg of each samples' total RNA was subsequently reverse transcribed using PrimeScript™ RT Reagent Kit with gDNA Eraser (Takara Bio Inc.). To generate a no amplification control (no amplification ctrl.), 1 µg SK-OV-3 RNA was used without addition of reverse transcriptase enzyme, replacing the enzyme's volume in the reaction with PCR-grade, RNase-free water. Expression of CLDN6 was determined via Droplet Digital™ PCR (ddPCR™) on a Bio-Rad QX200™ System. The ddPCR™ was performed with QX200™ ddPCR™ EvaGreen Supermix (BioRad) using the

following primers with a final concentration of 90 nM each in the reaction: *CLDN6* (forward 5'-CTT ATC TCC TTC GCA GTG CAG-3'; reverse 5'-AAG GAG GGC GAT GAC ACA GAG-3') *HPRT1* (forward 5'-TGA CAC TGG CAA AAC AAT GCA-3'; reverse 5'-GGT CCT TTT CAC CAG CAA GCT-3') [2]. The following thermal protocol was used in the PCR: Hot Start for 5 min at 95°C, 40 cycles of two-step PCR using a denaturation temperature of 95°C for 30 seconds and 62°C for 1 minutes for annealing/elongation. The last cycle was followed by a signal-stabilizing step (recommended by the manufacturer) with an incubation for 5 min at 4°C following 5 min at 95°C, finally the reaction was held at 12°C until the sample was transferred to the QX200™ Droplet Reader. All steps used a ramping rate of 2°C/sec. For each assay the positive threshold was defined individually, the total numbers of positive droplets were counted and *CLDN6* expression was normalized to reference gene *HPRT1*. Negative controls, no template control (no template ctrl.), no amplification control. Positive control, placenta sample.

References

[1] Schägger H, Jagow G von. Tricine-sodium dodecyl sulfate-polyacrylamide gel electrophoresis for the separation of proteins in the range from 1 to 100 kDa. Analytical Biochemistry 1987;166:368–79.

[2] Stadler CR, Bähr-Mahmud H, Celik L, Hebich B, Roth AS, Roth RP, et al. Elimination of large tumors in mice by mRNA-encoded bispecific antibodies. Nature medicine 2017;23:815–17.

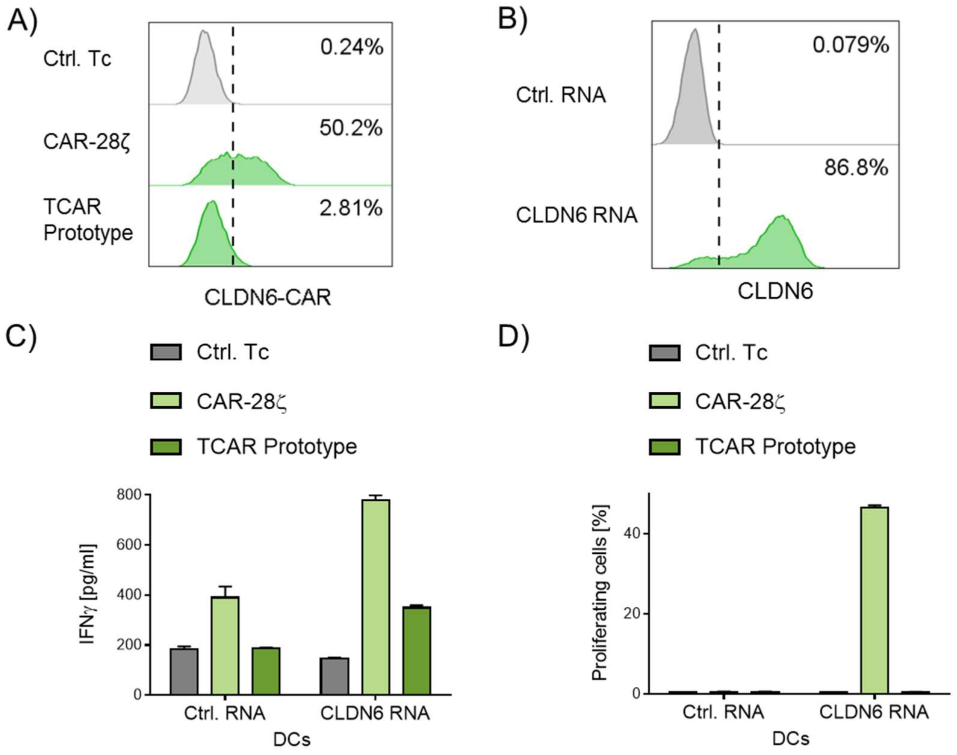

Fig. S1. CAR-28ζ is more effective than TCAR prototype at inducing IFNγ secretion and proliferation. **(A)** CAR surface expression on human $CD8^+$ T cells analyzed by flow cytometry. **(B)** CLDN6 surface expression on $CLDN6^+$ DCs analyzed by flow cytometry. **(C)** IFNγ concentrations in culture supernatants of co-cultures of CLDN6-CAR T cells with CLDN6-electroporated DCs (E:T ratio of 1:1). **(D)** Proliferation of receptor-transfected human $CD8^+$ T cells after coculture with antigen-transfected DCs (E:T = 6:1) analyzed by CFSE proliferation assay. T cells expressing TCAR prototype β-chain alone (Ctrl. Tc), and DCs expressing gp100 RNA (Ctrl. RNA) were used as controls. Graphs show mean + SD of technical duplicates.

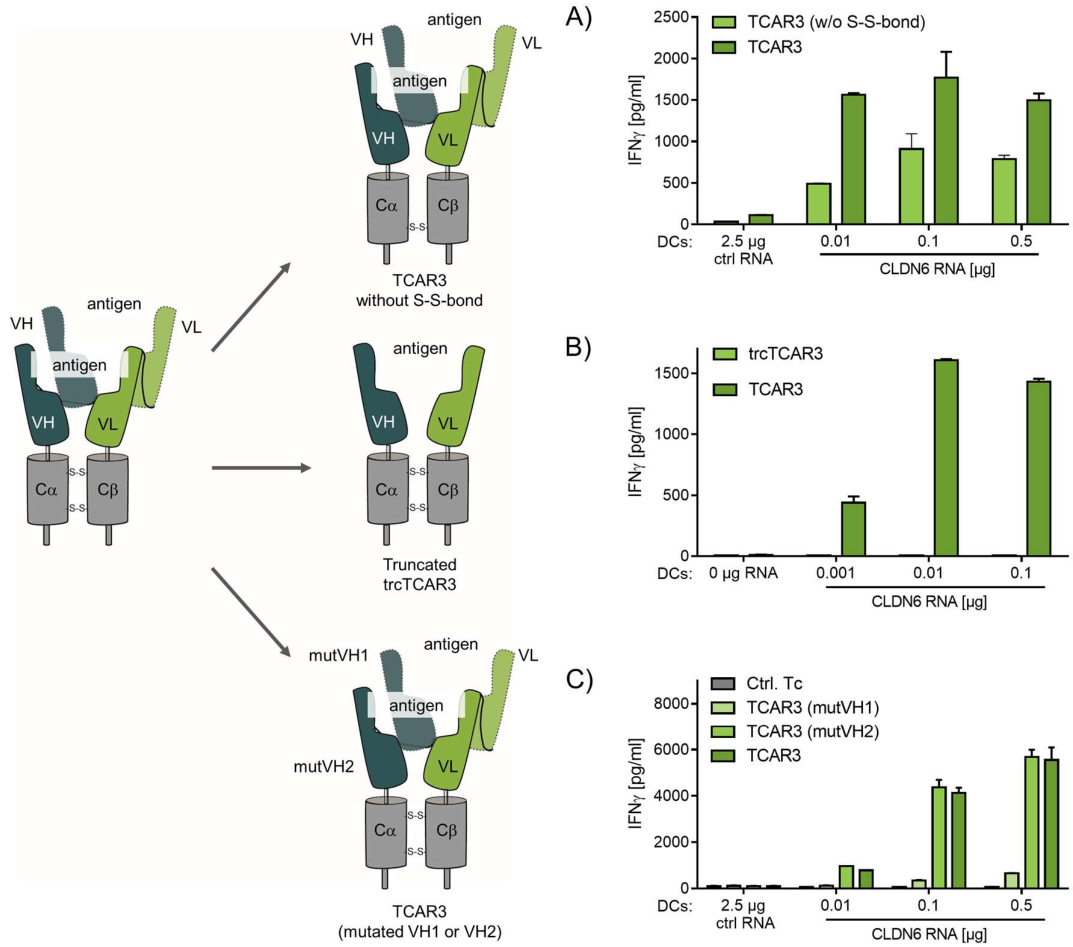

Fig. S2. Interaction between variable domains, rather than avidity, is required for TCAR3 function. Sequence variants of CLDN6-specific TCAR3 were tested for their impact on IFNγ secretion of human CD8$^+$ T cells upon antigen-specific stimulation with CLDN6$^+$ DCs. The three sequence variants are shown on the left. (**A**) Back-mutation of an artificial di-sulfide bond between the human constant domains of TCAR3. (**B**) Truncation (trc) of TCAR3 (with human constant domains) to only one pair of variable domains, and (**C**) Point mutations in the distal VH1 (mutVH1) or proximal VH2 (mutVH2) domains of TCAR3 with murine C-domains. DCs transfected with a control RNA (gp100 RNA (A), CLDN18.2 RNA (C)) or water (B), and T cells transfected with TCAR prototype α-chain alone (Ctrl. Tc) were used as negative controls. Graphs show mean + SD of technical duplicates.

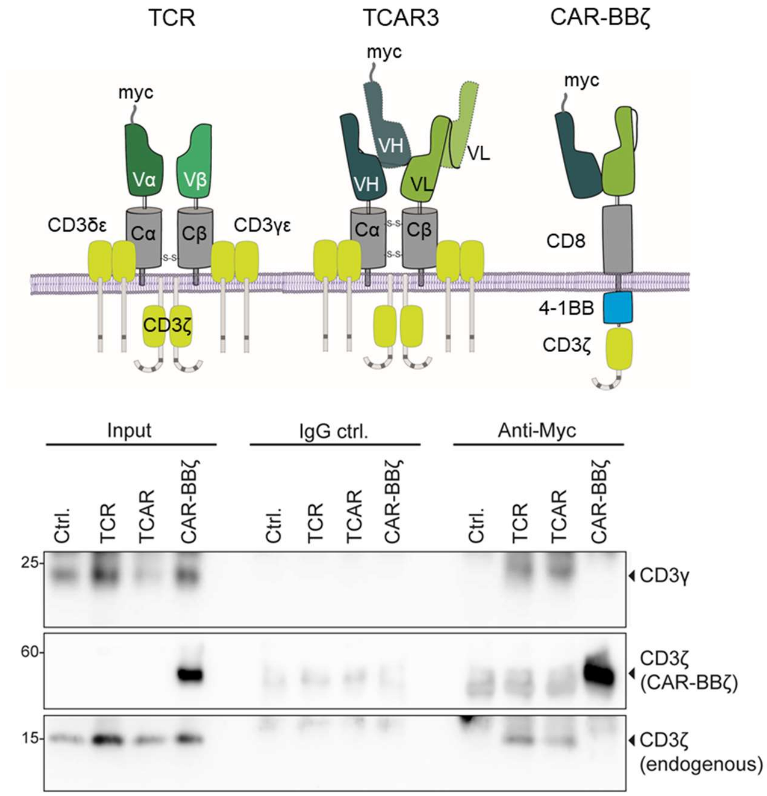

Fig. S3. The constant domains of TCAR3 recruit endogenous CD3. Co-immunoprecipitation of CD3 subunits with Myc-tagged mouse TCR, CAR-BBζ, or TCAR3 with mouse C-domains. Input: Cell lysates of receptor-transfected Jurkat76 cells. IgG ctrl.: pulldown control using mouse IgG isotype antibody. Anti-Myc: pulldown using anti-Myc antibody. Western blot shows antibody staining for co-precipitated CD3ζ and CD3γ chains. Jurkat76 transfected with Cα alone were used as a negative control (Ctrl.).

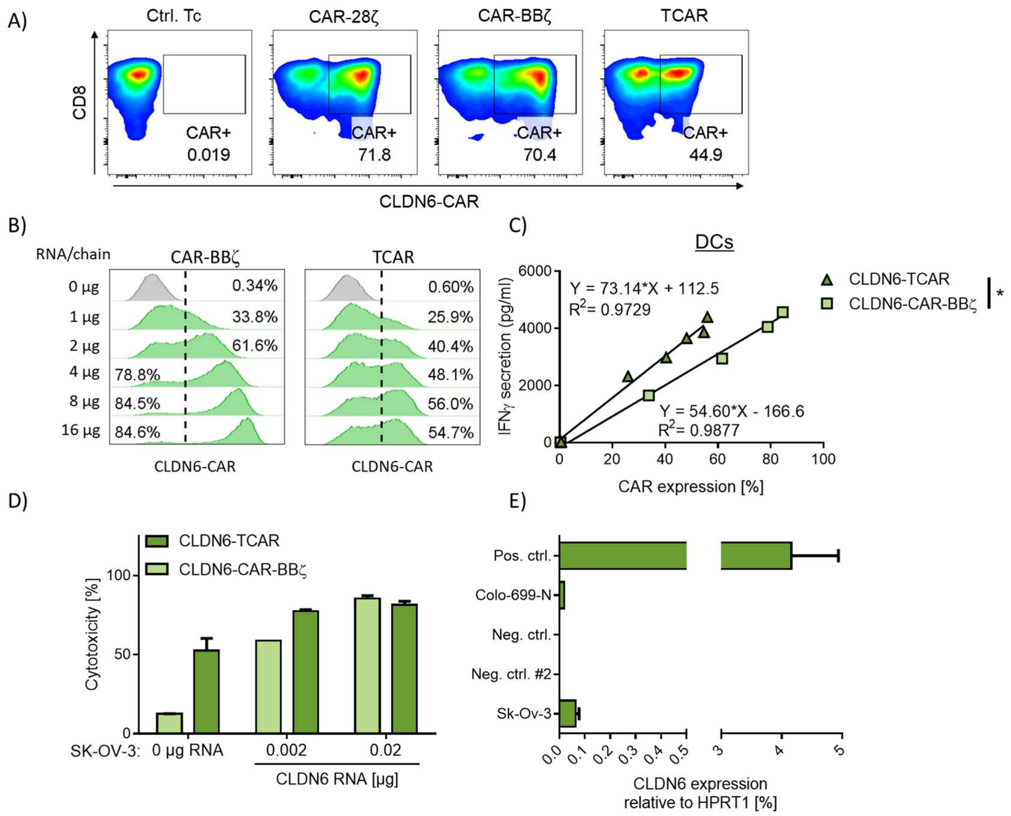

Fig. S4. CLDN6-TCAR surface expression in human CD8$^+$ T cells and recognition of CLDN6$^+$ target cells. (A, B) Receptor surface expression on human CD8$^+$ T cells transfected with receptor-encoding RNA analyzed by flow cytometry. Equimolar (30 pmol, with 20 µg CAR-28ζ, 15 µg CAR-BBζ and 12 µg TCAR) (A) and escalated (B) RNA amounts used per receptor-chain. Controls, Cα-transfected T cells (Ctrl.Tc). Mean + SD. **(C)** Correlation of receptor surface expression and cognate IFNγ secretion of receptor-transfected CD8$^+$ T cells in culture supernatants after co-culture with human CLDN6 RNA-transfected DCs (1 µg) for 20 h (E:T = 2:1). Unpaired *t*-test used to compare for statistical significance between slope of linear regressions in B. *$p < 0.05$. **(D)** Cytotoxicity of RNA-transfected T cells with equal CAR-T frequency (4 µg TCAR RNA and 1.75 µg CAR-BBζ RNA) against human SK-OV-3 tumor cells transfected with CLDN6 RNA or water (0 µg RNA) in xCELLigence-based cytotoxicity assay. **(E)** Expression level of CLDN6 in different cell types relative to HPRT by droplet digital PCR. Controls, no template control (Neg. ctrl.), no amplification control (Neg. ctrl. #2), placenta sample (pos. ctrl.).

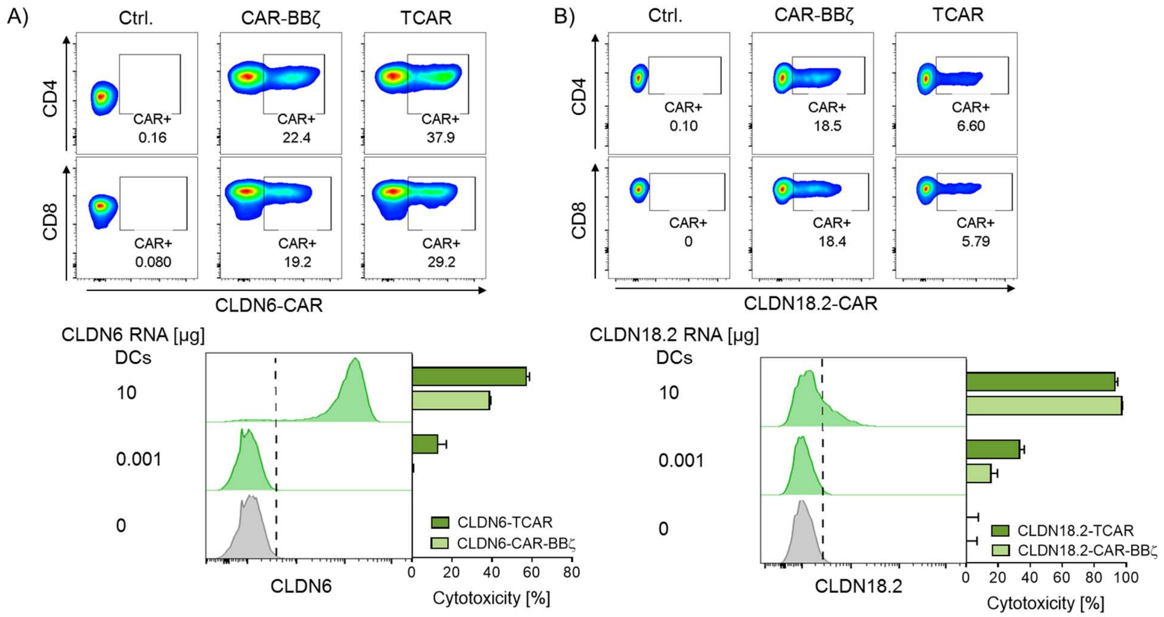

Fig. S5. T cells with stable TCAR expression generated by viral transduction show robust surface expression, and antigen-specific cytotoxicity. Characterization of CLDN6- (**A**) and CLDN18.2-TCAR (**B**) transduced $CD3^+$ human T cells, in comparison to CLDN6- and CLDN18.2-CAR-BBζ and negative controls. Controls, TCAR stained with isotype control antibody for CLDN6-CAR (Ctrl. in A) or non-transduced T cells stained with anti-CAR antibody (Ctrl. in B). (Top row) CLDN6- or CLDN18.2-CAR staining on receptor-expressing T cells, analyzed by flow cytometry. (Bottom row) Analysis of T cell-mediated recognition and cytotoxicity against $CLDN6^+$ or $CLDN18.2^+$ DCs after 24 h (E:T of 6:1). CAR-T frequencies were not adjusted for this assay. Graphs show mean + SD of technical triplicates.

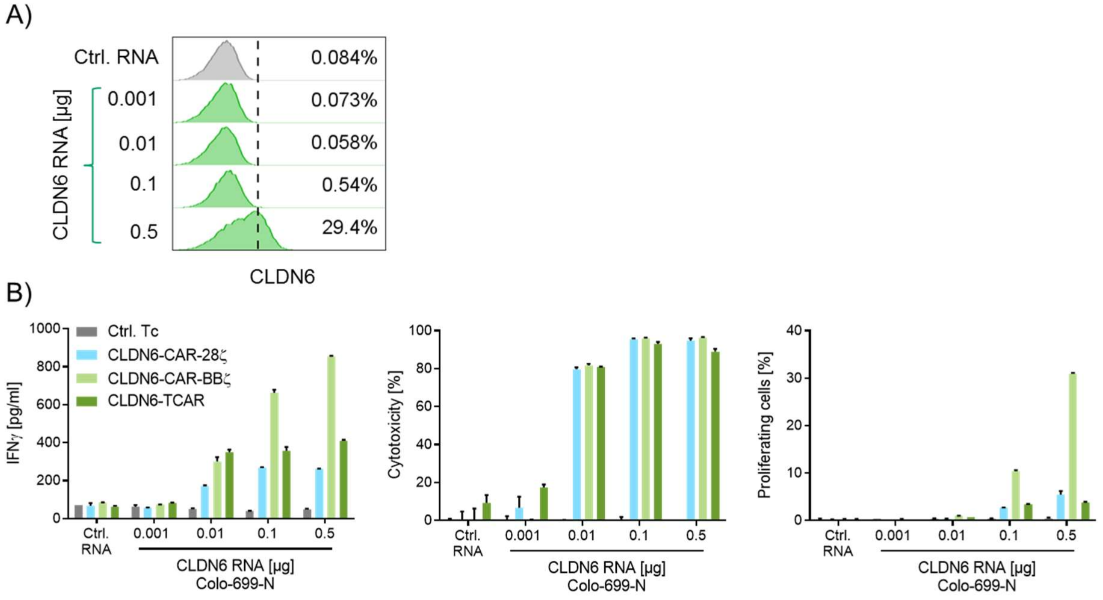

Fig. S6. TCAR T cells mount potent effector functions but lack proliferation against tumor cells. **(A)** CLDN6 surface expression on CLDN6 RNA-transfected human Colo-699-N cells analyzed with flow cytometry. Colo-699-N cells transfected with 2.5 µg CLDN18.2 RNA (Ctrl. RNA) were used as a negative control. **(B)** IFNγ secretion (left), cytotoxicity (middle) and proliferation (right) of equimolar receptor RNA-transfected human $CD8^+$ T cells co-cultured (expression data in Fig. S4A) with CLDN6-transfected Colo-699-N (E:T ratio = 10:1 or 20:1 (right)). IFNγ was analyzed by IFNγ ELISA, cytotoxicity was assessed using a luciferase-based cytotoxicity assay, and proliferation was assessed using a CFSE-mediated proliferation assay. T cells transfected with Cα RNA only (ctrl. Tc), and Colo-699-N cells transfected with CLDN18.2 RNA (Ctrl. RNA) were used as negative controls. Graphs show mean + SD of technical duplicates (left, right) or triplicates (middle) representative for 3 blood donors.

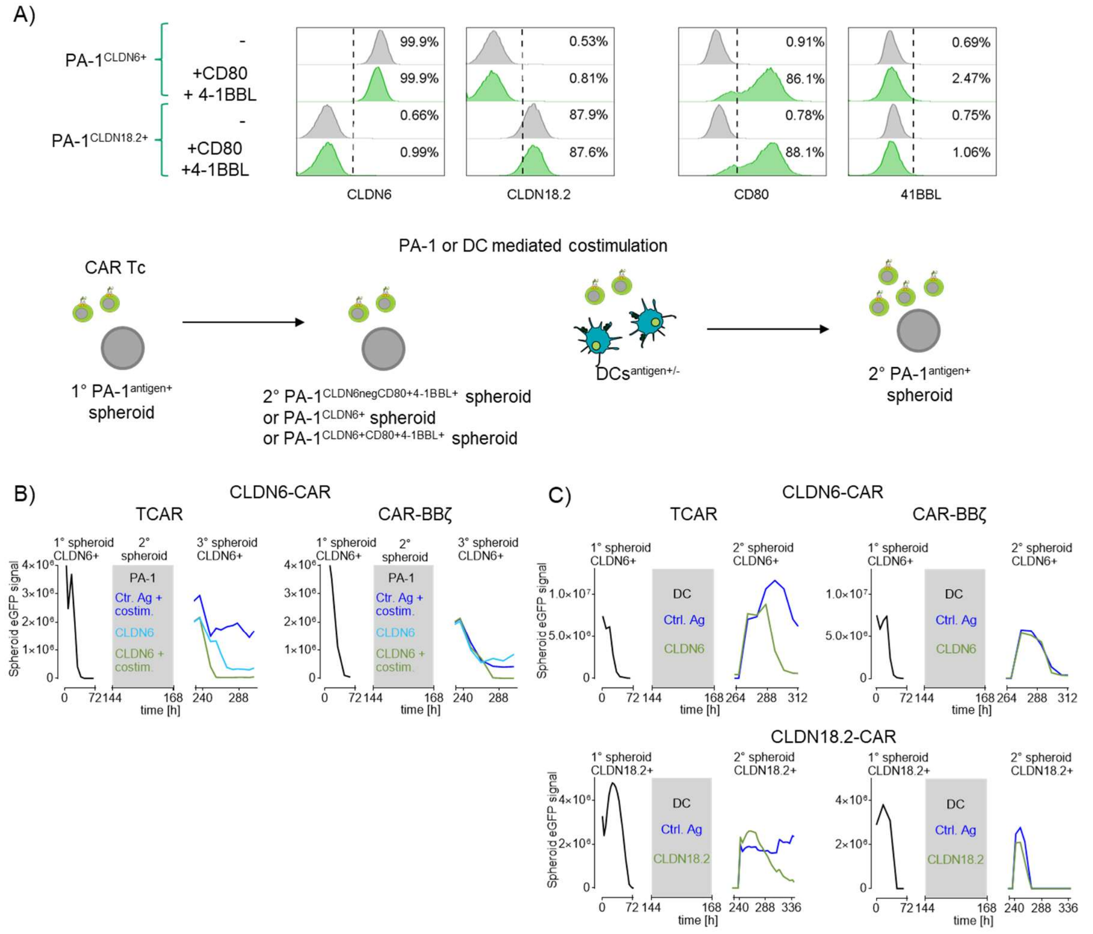

Fig. S7. Antigen-specific stimulation of CLDN6 and CLDN18.2-CAR T cells via CD80 and 4-1BBL transfected PA-1 spheroids or DCs enhances subsequent tumor spheroid killing. Serial killing of human eGFP$^+$ CLDN$^+$ PA-1 spheroids by human receptor-transduced T cells monitored by *in vitro* live cell imaging of eGFP$^+$ tumor cells. **(A)** CLDN6, CLDN18.2, CD80 and CD137L staining on transfected PA-1 tumor cells. **(B)** Repetitive tumor spheroid assay with tumor cell-mediated costimulation phase (grey inlay). CLDN6-specific CAR T cells were co-cultured with: (1°) PA-1^{CLDN6+} spheroids (E:T 30:1); (2°, grey inlay) PA-1^{CLDN6+} spheroids transfected with costimulatory molecules CD80 and 4-1BBL; (3°) PA-1^{CLDN6+} spheroids. PA-1$^{CLDN18.2+CD80+4\text{-}1BBL+}$ served as negative control (Ctrl. Ag$^{costim+}$). **(C)** Repetitive tumor spheroid assay with DC-mediated costimulation phase (grey inlay). CLDN6- (upper panel) or CLDN18.2-specific (lower panel) CAR T cells co-cultured with: (1°) PA-1^{CLDN6+} or PA-1$^{CLDN18.2+}$ tumor spheroids (E:T 30:1 or 10:1, respectively); (grey inlay) CLDN$^+$ DCs for 5 days; (2°) PA-1^{CLDN6+} and PA-1$^{CLDN18.2+}$ tumor spheroids. Controls, CLDN18.2$^+$ DCs served as negative controls for CLDN6-CAR (Ctrl. Ag) and vice versa. Graphs show mean CAR T cell killing kinetics of three (A, B, upper panel) or four (B, lower panel) technical replicates.

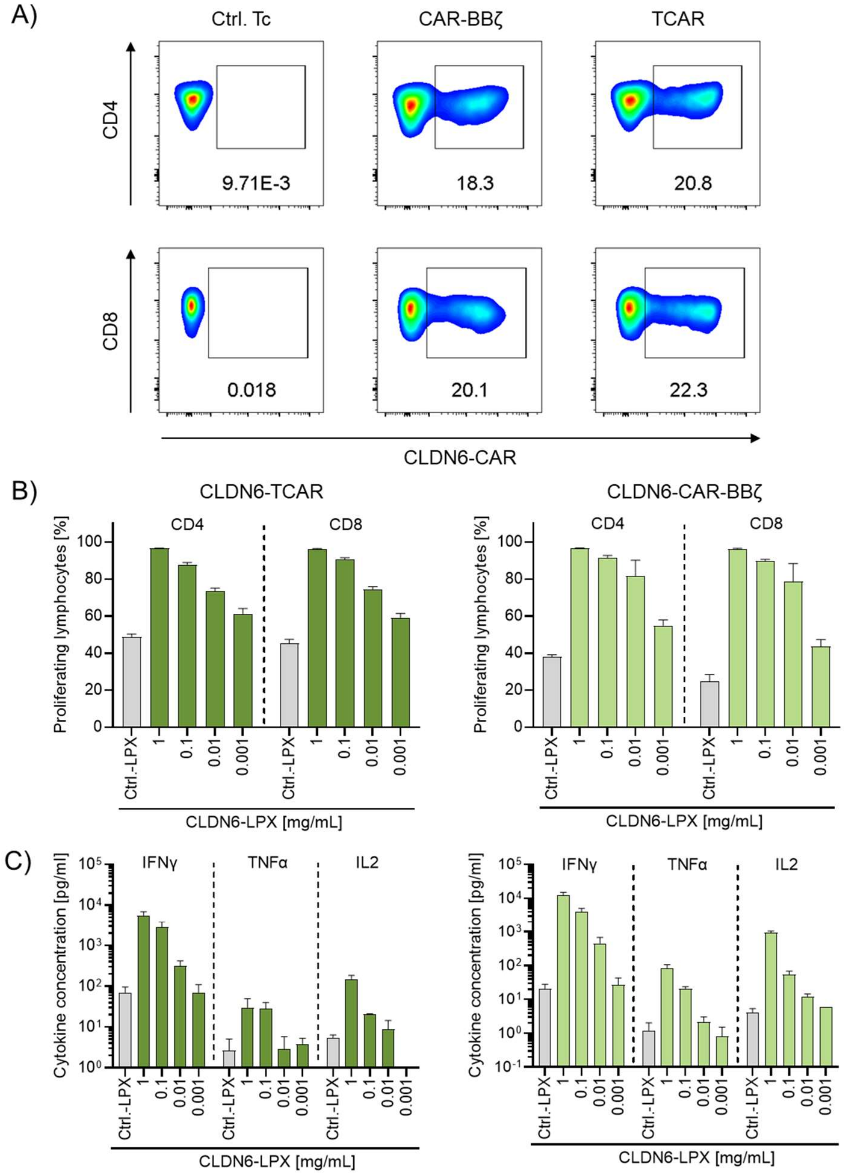

Fig. S8. CLDN6-TCAR and CAR-BBζ T cells proliferate and secrete cytokines against CLDN6-LPX treated DCs. (A) Expression of CLDN6-TCAR/CAR on T cells after retroviral transduction. **(B)** Proliferation and **(C)** cytokine secretion of human CLDN6-TCAR (left) or CLDN6-CAR-BBζ (right) transduced T cells after co-culture with RNA-LPX treated DCs (E:T 10:1). Proliferation of CFSE labeled T cells was assessed using flow cytometry. Cytokine secretion was analyzed via a multiplex assay. DCs treated with 1 mg/mL eGFP RNA-LPX served as negative control (Ctrl.-LPX). Graphs show mean + SD of technical triplicates.

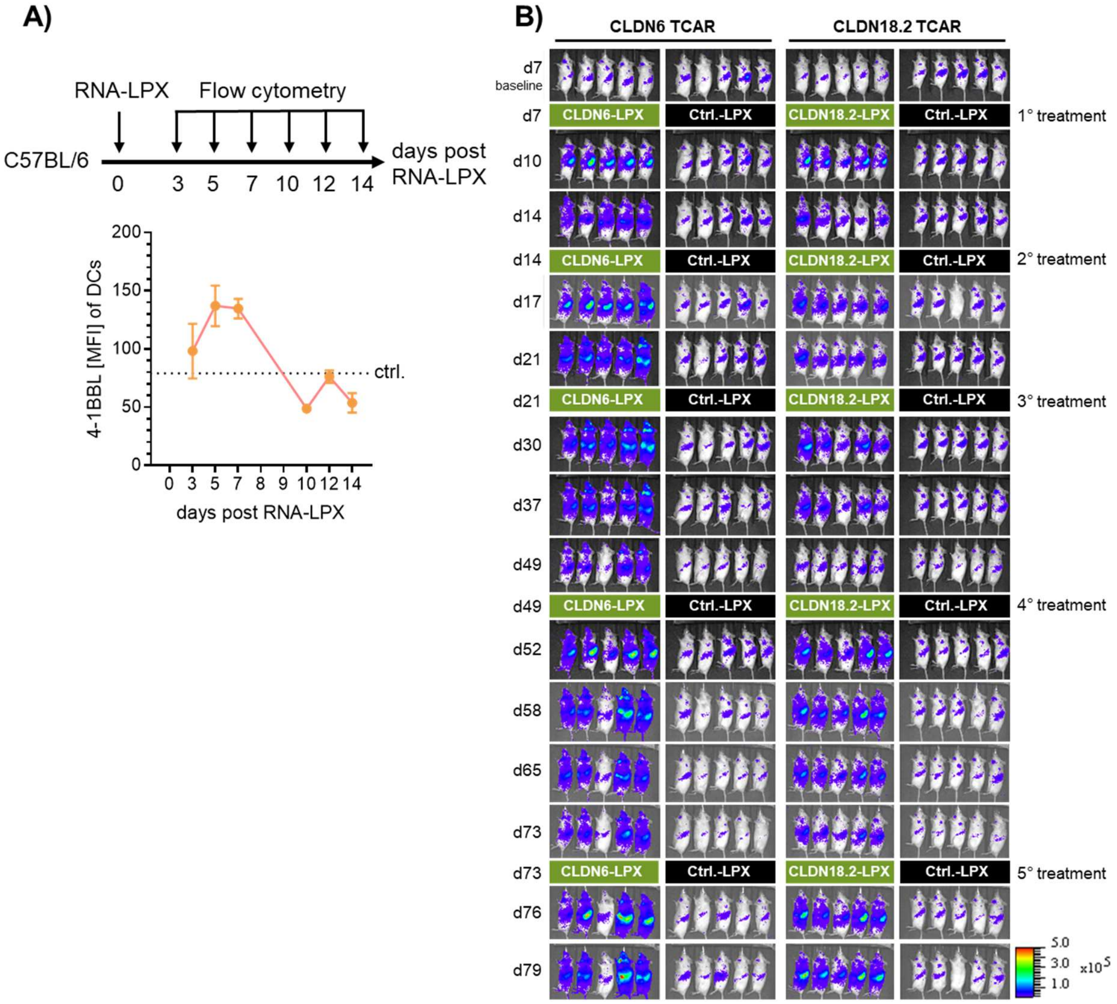

Fig. S9. RNA-LPX facilitates upregulation of costimulatory molecules in splenic DCs and expands TCAR-T antigen-specifically. (A) 4-1BBL expression kinetics on splenic DCs after RNA-LPX treatment (20 µg $OVA_{257\text{-}264}$) detected as mean fluorescence intensity (MFI) via flow cytometry. Sodium chloride was administered as treatment control (ctrl.). **(B)** Luc-GFP-expressing $Thy1.1^+$ CLDN6- or CLDN18.2-TCAR mouse T cells were transferred into total body irradiated (TBI) $Thy1.2^+$ C57BL/6-albino mice (n = 5 mice/group). Mice were treated with 20 µg of CLDN6 or CLDN18.2 encoding RNA formulated into lipoplexes. Bioluminescence imaging of mice was monitored over 79 days. CLDN18.2-LPX served as negative control (Ctrl. LPX) for CLDN6-CARs, and CLDN6-LPX as a negative control for CLDN18.2-CARs. Images correspond to data presented in Fig. 2C.

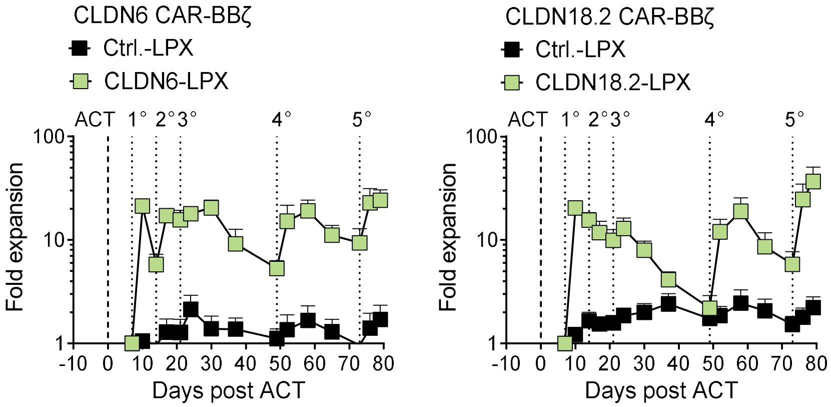

Fig. S10. RNA-LPX mediated *in vivo* expansion of CAR-BBζ engineered murine T cells. RNA-LPX vaccination mediates CAR-BBζ T cell expansion in a syngeneic *in vivo* mouse model. Luc-GFP-expressing Thy1.1^{+} CLDN6- or CLDN18.2-CAR-BBζ mouse T cells were transferred into total body irradiated (TBI) Thy1.2^{+} C57BL/6-albino mice (n = 5 mice/group). Seven days later mice were injected with 20 µg CLDN6 or CLDN18.2 RNA-LPX. Kinetics of CAR-BBζ T cell expansion by bioluminescence imaging (BLI, upper panel) are shown. CLDN18.2-LPX served as negative control (Ctrl. LPX) for CLDN6-CARs, and CLDN6-LPX as a negative control for CLDN18.2-CARs. Mean + SEM of 5 mice per group is shown.
